# Itaconic Acid as a Comonomer in Betulin-Based Thermosets via Sequential and Bulk Preparation

**DOI:** 10.1021/acssuschemeng.3c04178

**Published:** 2023-09-13

**Authors:** Alexandra M. Lehman-Chong, Casey L. Cox, Emre Kinaci, Sarah E. Burkert, Megan L. Dodge, Devin M. Rosmarin, James A. Newell, Lindsay Soh, Melissa B. Gordon, Joseph F. Stanzione

**Affiliations:** † Department of Chemical Engineering, Rowan University, 201 Mullica Hill Road, Glassboro, New Jersey 08028, United States; ‡ Advanced Materials & Manufacturing Institute (AMMI), Rowan University, 201 Mullica Hill Road, Glassboro, New Jersey 08028, United States; § Department of Chemical and Biomolecular Engineering, Lafayette College, 740 High Street, Easton, Pennsylvania 18042, United States

**Keywords:** betulin, itaconic acid, unsaturated polyesters, biobased, thermoplastics, thermosets, photopolymerization, methacrylates

## Abstract

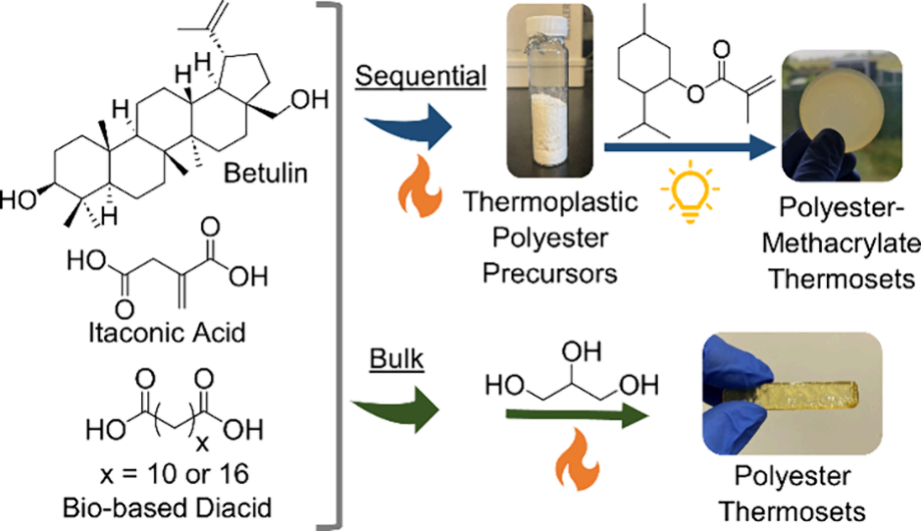

The inherent chemical functionalities of biobased monomers enable the production of renewably sourced polymers that further advance sustainable manufacturing. Itaconic acid (IA) is a nontoxic, commercially produced biobased monomer that can undergo both UV and thermal curing. Betulin is a biocompatible, structurally complex diol derived from birch tree bark that has been recently studied for materials with diverse applications. Here, betulin, IA, and biobased linear diacids, 1,12-dodecanedioic acid (C12) and 1,18-octadecanedioic acid (C18), were used to prepare thermosets using sequential and bulk curing methods. Thermoplastic polyester precursors were synthesized and formulated into polyester-methacrylate (PM) resins to produce sequential UV-curable thermosets. Bulk-cured polyester thermosets were prepared using a one-pot, solventless melt polycondensation using glycerol as a cross-linker. The structure–property relationships of the thermoplastic polyester precursors, sequentially prepared PM thermosets, and bulk-cured polyester thermosets were evaluated with varying IA content. Both types of thermosets exhibited higher storage moduli, *T*_g_s, and thermal stabilities with greater IA comonomer content. These results demonstrate the viability of using IA as a comonomer to produce betulin-based thermosets each with tunable properties, expanding the scope of their applications and use in polymeric materials.

## Abstract 2

Thermoplastic polyester precursors were synthesized and formulated into polyester-methacrylate (PM) resins to produce sequential UV-curable thermosets. Bulk-cured polyester thermosets were prepared using a one-pot, solventless melt polycondensation using glycerol as a cross-linker.

## Introduction

Biobased products are of growing interest due to the need for sustainable manufacturing, the desire to reduce dependency on nonrenewable petroleum, and the opportunity to increase economic activity and job growth for a developing bioeconomy.^1−3^ The environmental and health impacts of chosen monomers and chemical additives are increasingly important to consider when designing new polymers as chemical leaching can result in negative human and environmental exposure.^4,5^ Thus, it is important to intentionally consider not only the renewable origins but also the impacts of chosen monomers in biobased polymer design throughout all stages of the polymer life cycle including feedstock sourcing, manufacturing, use, and end-of-life.^4^ In addition to safety and environmental impacts, the inherent chemical functionalities of biobased monomers are important to consider. The versatility of these functionalities enables the production of diverse biobased polymers that expand the scope of applications, which helps promote more widespread use.

Itaconic acid (IA) is an example of a dual-functional, biocompatible biobased monomer with increasing use as it is commercially available from fermented agricultural waste and is projected to reach a market of 177 million USD by 2028.^6,7^ As an unsaturated diacid monomer, IA can undergo both polycondensation and free radical polymerization, allowing for its use in diverse polymers. Such polymers include thermoplastic and thermoset polyesters, with applications in textiles, shape memory polymers, coatings, UV-curable additive manufacturing, and biomedicine.^6,8−13^

Betulin is another versatile, structurally complex biobased monomer that can be extracted from the bark of birch trees at quantities up to 30 dry wt %.^14^ It is a pentacyclic triterpenoid with a fused cycloaliphatic and chiral structure. This structural complexity has inspired the incorporation of betulin and its derivatives into a range of novel materials used for applications such as hydrogels, electro-optically responsive materials, UV-stable materials, and hierarchical nano- and microstructures.^15−21^ The breadth of recently reported studies that utilize the structural features of betulin for diverse applications highlights the timely relevance of betulin as a biobased molecule in materials research. Additionally, betulin exhibits natural therapeutic activity and, therefore, may be a promising candidate for use in biocompatible and nontoxic polymers.^22−30^

Previously, we synthesized betulin-based polyester thermoplastics and thermosets with tunable high-performance properties using a series of linear aliphatic diacids.^31^ Recently, Papadopoulos et al. reported the synthesis of thermoplastic polyesters from IA with varying linear aliphatic and aromatic diacid comonomers cured with acrylate reactive diluents for additive manufacturing (AM) materials.^32^ Shivarkar et al. used IA to synthesize unsaturated polyesters that were then grafted with acrylic monomers to produce coatings with improved mechanical properties, adhesion, and weathering resistance.^33^ Dai et al. used IA as a diacid comonomer to improve the high-performance properties of soybean oil-based thermosets for coatings applications.^34^ Additionally, IA and polyols have been used to synthesize branched polymer networks for UV-curable biomedical applications.^35^ Drawing inspiration from these studies, we aim to expand the diversity of betulin-based polymer applications by utilizing the versatility of IA as a comonomer to synthesize thermosets. Thermosets offer critical improvements in mechanical properties over thermoplastics, are advantageous for certain biomedical applications that require the ability to retain 3D structures, and are generally more suitable for certain resin-based AM techniques.^35^

Here, to utilize both the diacid and unsaturated functionalities of IA, we explored two procedures for synthesizing different betulin-based thermosets via sequential and bulk curing methods. These methods were chosen to highlight the potential for IA in furthering the tunability and scope of the betulin-based polyester properties. First, unsaturated betulin-based thermoplastic polyesters were synthesized with IA via a melt polycondensation technique and used as precursors for sequentially preparing UV-curable polyester-methacrylate (PM) thermosets. This approach avoided limitations of direct betulin (meth)acrylation, including multistep synthesis and purification procedures using hazardous reagents, and also allowed for the evaluation of thermoplastic precursors prior to controlled sequential curing.^36−38^ Next, we synthesized betulin-based polyester thermosets with a bulk melt polycondensation procedure using IA as a diacid comonomer and glycerol as a cross-linker. This method allowed for a one-pot thermal curing approach that incorporated betulin into cross-linked networks without the need for any prior monomer functionalization. For thermosets prepared by using either approach, the IA content was varied to study the compositional effects on the properties of the resulting thermosets.

Ultimately, this work focuses on developing betulin-based thermoset networks by using IA as a comonomer through two different synthetic approaches that possess potential trade-offs in life cycle impacts, specifically during production and end-of-life phases. Furthermore, by selecting biorenewable, biocompatible IA and betulin as monomers, we aimed to strategically design polymer networks that may mitigate negative environmental and health impacts throughout the polymer life cycle. The results presented herein highlight the versatility of these monomers and expand the scope of applications for betulin-based polymers, showing that each thermoset preparation method is viable depending on the desired processing conditions, properties, and final application.

## Experimental Section

### Materials

Betulin (98%) was purchased from BOC Sciences. 1,18-octadecanedioic acid (C18, >98%), 1,12-dodecanedioic acid (C12, >99%), dimethyl itaconate (DMI, >98%), diphenyl(2,4,6-trimethylbenzoyl)phosphine oxide (TPO, >98%), menthol (>98%), and dibutyltin dilaurate (>95.0%, stored at 5 °C) were purchased from TCI America. Itaconic acid (IA, >99%), chloroform-*d*_3_ (CDCl_3_, >99.8%), dibutyltin oxide (DBTO, 98%), 4-dimethylaminopyridine (4-DMAP, 99%), and hydroquinone (99%) were purchased from Acros Organics. Glycerol (≥99.5%) was purchased from Sigma-Aldrich. Methanol (≥99.8%), dichloromethane (DCM, ≥99.5%), ethyl acetate (≥99.5%), sodium hydroxide (≥98%), sodium chloride (≥99.0%), and tetrahydrofuran (THF, HPLC grade) were purchased from VWR. Methacrylic anhydride (94%, stabilized with ca. 0.2% 2,4-dimethyl-6-*tert*-butylphenol) was purchased from Alfa Aesar. Magnesium sulfate was purchased from Thermo Scientific. All chemicals mentioned above were used as received and stored at room temperature unless otherwise noted. A Miller-Stephenson PTFE release agent (MS-122) was acquired from Grainger. Nitrogen (N_2_, 99.999%), argon (Ar, 99.998%), and liquid nitrogen (LN_2_, 99.998%) were purchased from Airgas and used as received.

### Synthesis of Thermoplastic Polyester Precursors

**Scheme 1:**
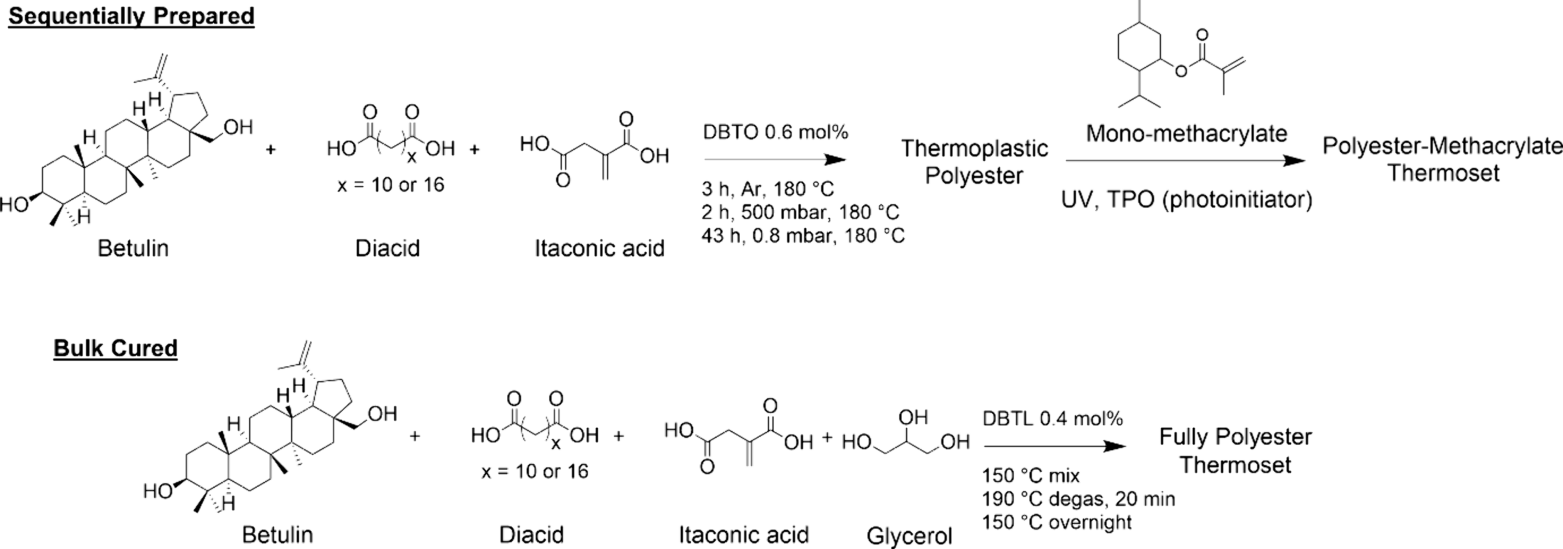
General Reaction Schemes for the Sequentially Prepared and Bulk-Cured Thermosets

A series of betulin-based thermoplastic polyesters were synthesized with C12, C18, and IA in varying molar ratios using a procedure adapted from Curia et al. (Scheme 1).^31^ Further details and ^1^H NMR and ^13^C NMR spectra of the synthesized polymers are listed in the Supporting Information (SI).

### Synthesis of the Menthyl Methacrylate (MenMA) Monomer

Menthyl methacrylate (MenMA) was synthesized from menthol and used as a biobased monofunctional methacrylate monomer for polyester-methacrylate (PM) resins. Synthesis details are found in the SI.

### Polyester-Methacrylate (PM) Resin Preparation and Curing

Polyester-methacrylate (PM) resins were prepared by adding thermoplastic polyesters with and without IA at varying wt % loadings to MenMA. In general, MenMA, the chosen thermoplastic polyester, TPO (1 wt % of total), and a magnetic stirrer were added to an amber scintillation vial, capped, and stirred until the mixture was homogeneous in a 60 °C water bath. PM resins were then poured into aluminum pans, placed in a Formlabs Form Cure (39 W LED power, 405 nm light) preheated to 50 °C, and left to cure for 4 h. The final PM polymers were left to cool to ambient temperature, cut, and sanded to appropriate dimensions for property characterizations.

### Synthesis of Bulk-Cured Polyester Thermosets

Betulin-based polyester thermosets were synthesized with C12, C18, and IA in varying molar ratios with glycerol as a cross-linker using a melt polycondensation procedure (Scheme 1).^31^ In general, the chosen total diacid (12.2 mmol total) was first fully melted in a 20 mL scintillation vial. Then, betulin (2.6 mmol, 1.13 g), glycerol (6.4 mmol, 0.59 g), and dibutyltin dilaurate (∼0.4 mol %, 47 μL) were sequentially added while stirring. The solution was stirred until it was homogeneous and reacted at 150 °C until a qualitative increase in viscosity was observed. For each composition, mixing times were established based on preliminary experiments that determined times for complete gelation. Mixing times for compositions with IA were 5 h for C12 and 6 h for C18. Mixing times for compositions without IA were 10 h for C12 and 13 h for C18. The viscous mixture was then transferred into PTFE molds (44.5 mm × 8 mm × 1.7 mm) that were preheated to 150 °C and pretreated with the PTFE release agent. The specimens were degassed at 190 °C for 20 min and then cured at 150 °C overnight. After being cooled to room temperature, the samples were removed and cut to proper dimensions for testing.

### Characterization Methods

Details regarding the characterization methods are found in the SI.

## Results and Discussion

### Sequentially Prepared Polyester-Methacrylate (PM) Thermosets

#### Thermoplastic Polyester Precursor Synthesis and Characterization

Betulin-based thermoplastic polyesters were synthesized using equimolar ratios of betulin to total diacid. A 50/50 molar ratio of linear aliphatic diacid (C12 or C18) to IA was used to synthesize thermoplastic polyester precursors for preparing sequential thermosets, and thermoplastics without IA were synthesized for comparison. Linear aliphatic diacids, C12 and C18, were chosen to provide reaction mixtures with relatively low melt viscosities, which enabled better mixing and controlled reaction conditions compared to diacids with shorter chain lengths. Thermoplastic polyester precursors containing 50/50 diacid/IA are denoted as C12/IA-TP and C18/IA-TP, and thermoplastics without IA are denoted as C12-TP and C18-TP.

Incorporation of IA into the thermoplastic polyester precursors was confirmed by ^1^H NMR and ^13^C NMR spectroscopic analyses. From a representative ^1^H NMR spectrum of C12/IA-TP (Figure S5), we observed characteristic peaks for IA around 6.41, 5.79, and 3.37 ppm. Peak splitting of these signals can be attributed to the different ester bonds formed between asymmetric betulin and IA monomers.^8,39^ Furthermore, ^13^C NMR spectra show the successful formation of ester bonds from betulin with both the long-chain diacid and IA (Figures S6 and S8). Other assigned ^1^H NMR and ^13^C NMR peaks are attributed to betulin and either the C12 or C18 diacid. A small ^1^H NMR peak at 6.77 ppm shows that there was a slight rearrangement of IA to mesaconate, and a small broad peak between 2.5 and 3.0 ppm indicates that slight branching occurred due to the Ordelt reaction; however, these signals were small relative to the peaks from IA, and related signals could not be seen in ^13^C NMR.^40^ These side reactions are frequently mitigated by incorporating an inhibitor during thermoplastic polyester synthesis while using IA.^32,41,42^ Here, the extents of IA isomerization and branching were mitigated by the presence of a long aliphatic diacid, similar to a reported study by Farmer et al. that utilized succinic acid as a comonomer.^40^ Using this approach, thermoplastics were also synthesized with varying degrees of IA content to investigate the tunability of thermoplastic polyester precursor properties. ^1^H NMR and ^13^C NMR spectroscopic analyses were conducted for all thermoplastic polyester precursor compositions described herein, including a thermoplastic homopolymer synthesized from betulin and dimethyl itaconate, poly(betulin-dimethyl itaconate) (BDMI) (see the SI). Although the addition of C12 and C18 mitigated IA side reactions, thermoplastics with higher IA content exhibited more prevalent side products. These additional thermoplastics were characterized but not used as precursors for thermoset preparation.

**Figure 1:**
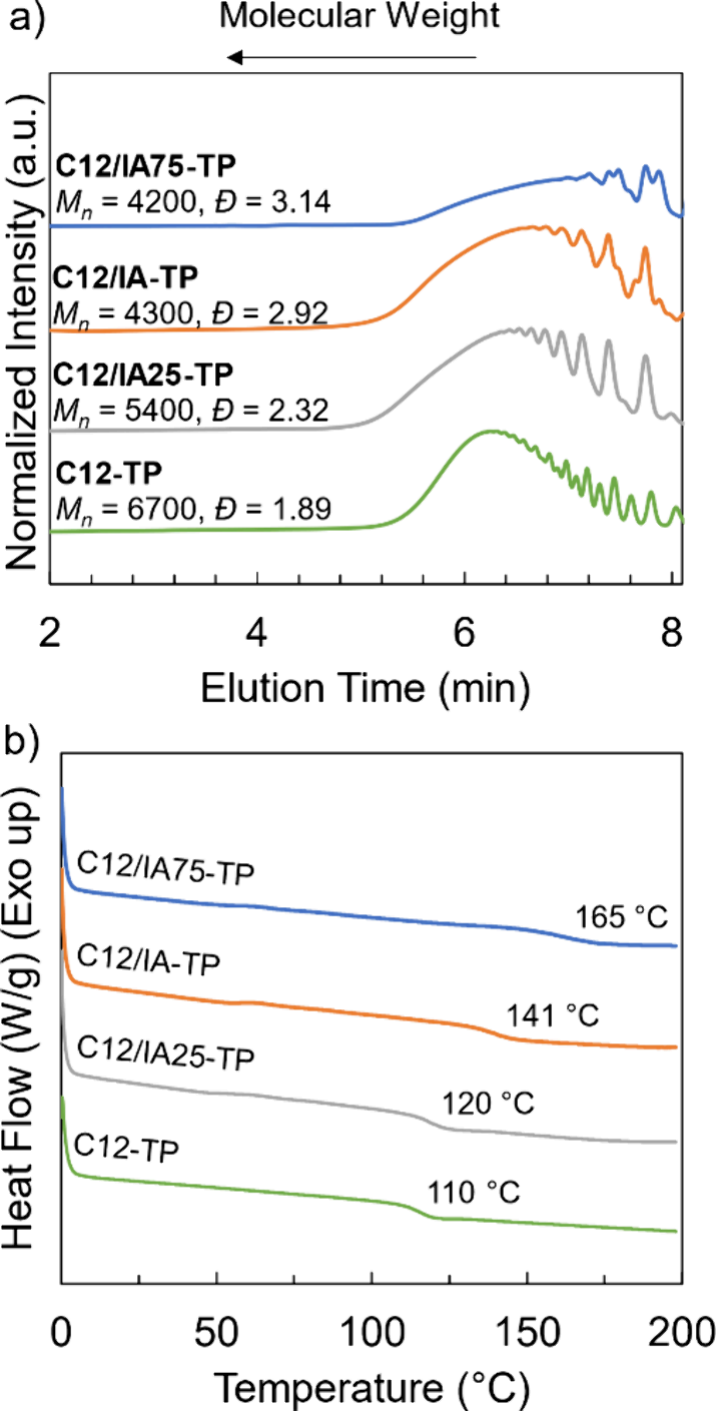
Representative (a) APC and (b) DSC traces from the 2nd heating ramp of thermoplastic polyesters synthesized with 25/75 (C12/IA75-TP), 50/50 (C12/IA-TP), 75/25 (C12/IA25-TP), and 100/0 (C12/IA-TP) mol diacid/IA. Traces are normalized and stacked for clarity.

The molecular weight distributions of the synthesized thermoplastics were determined by using APC (Table 1). The number-average molecular weight (*M*_n_) and weight-average molecular weight (*M*_w_) values decreased with increasing IA content for both C12- (Figure 1) and C18-based thermoplastics (Figure S25), and overall, the C18-based thermoplastics possessed higher molecular weights due to the higher carbon chain length of the C18 diacid. The dispersity values of the thermoplastics increased with increasing IA content, which may be due to the more prevalent side reactions qualitatively observed in the NMR spectra. From differential scanning calorimetry (DSC) measurements, both C12- and C18-based thermoplastics exhibited an increasing glass transition temperature (*T*_g_) with increasing IA content, likely due to the shorter chain length of IA (Figure 1 and Figure S26). The compiled results in Table 1 indicate that C12-based thermoplastic polyesters exhibited *T*_g_ values ranging from 110 to 165 °C, which were higher than *T*_g_s of the longer-chain C18-based thermoplastic polyesters (76 to 134 °C). A clear absence of melting behavior from the first DSC heating ramps indicated that these thermoplastics were amorphous (Figure S27). Due to the shorter diacid chain length, BDMI displayed the highest *T*_g_ with a value of 170 °C.

**Table 1:** Molecular Weight Distributions and Thermal Data for Synthesized Thermoplastic Polyesters

**thermoplastic polyester**	**diacid/IA**(mol/mol)	***M*_n_**(g mol^–1^)	***M*_w_**(g mol^–1^)	***Đ***	***T*_g,DSC_(°C)**	**N**_**2**_		**air**	
						**IDT (°C)**	*T***_50_(°C)**	**IDT (°C)**	*T***_50_(°C)**
C12-TP	100/0	6700 ± 600	12600 ± 800	1.89 ± 0.10	110 ± 2	393 ± 5	437 ± 5	392 ± 12	430 ± 5
C12/IA25-TP	75/25	5400 ± 300	12500 ± 800	2.32 ± 0.04	120 ± 4	381 ± 6	434 ± 3	382 ± 1	434 ± 1
C12/IA-TP	50/50	4300 ± 600	12700 ± 3700	2.92 ± 0.64	141 ± 5	367 ± 7	437 ± 7	358 ± 8	442 ± 9
C12/IA75-TP	25/75	4200 ± 1300	14100 ± 10400	3.14 ± 1.48	165 ± 7	321 ± 21	412 ± 12	312 ± 21	420 ± 15
C18-TP	100/0	9300 ± 800	20500 ± 1800	2.19 ± 0.15	76 ± 2	390 ± 4	435 ± 5	382 ± 8	426 ± 2
C18/IA25-TP	75/25	6800 ± 1400	17600 ± 5900	2.55 ± 0.63	93 ± 4	396 ± 5	445 ± 9	379 ± 17	436 ± 6
C18/IA-TP	50/50	4400 ± 300	11300 ± 2300	2.56 ± 0.33	111 ± 5	373 ± 6	444 ± 2	363 ± 6	438 ± 3
C18/IA75-TP	25/75	3600 ± 500	12500 ± 6500	3.37 ± 1.31	134 ± 2	305 ± 31	410 ± 20	312 ± 33	427 ± 13
BDMI	0/100	3300 ± 300	5200 ± 600	1.57 ± 0.08	170 ± 8	298 ± 6	370 ± 3	296 ± 8	396 ± 7

Thermogravimetric analysis (TGA) was conducted to evaluate the thermal stability of these thermoplastic polyesters. General decreases in the initial decomposition temperature (IDT) and temperature of 50% mass loss (*T*_50_) were observed in both inert (N_2_) and oxidative (air) environments with increasing IA content for all thermoplastics (Table 1). This decrease in thermal stability is correlated with the lowered molecular weight of the polymer backbone and with increased IA content. IDT and *T*_50_ values appeared similar comparing C12-based versus C18-based thermoplastics and were also similar under N_2_ versus air environments, as previously reported.^31^

#### Polyester-Methacrylate (PM) Thermoset Properties

To prepare thermoset networks sequentially from the thermoplastic polyester precursors, we formulated UV-curable polyester-methacrylate (PM) resins with both C12/IA-TP and C18/IA-TP at varying weight percentages and evaluated the resulting structure–property relationships. PM resins with C12-TP and C18-TP thermoplastics were prepared for comparison.

We first synthesized a biobased monomethacrylate monomer from menthol, menthyl methacrylate (MenMA). Studies have reported that MenMA possesses inherent chirality, interesting optical properties, and potential as a biobased reactive diluent as it is less volatile than styrene, which is a suspected human carcinogen.^43−45^ Like betulin, MenMA is cycloaliphatic, which makes it suitable for creating thermosets that maintain fully aliphatic polymer networks with potentially high UV stability.

Next, thermoplastic polyester precursors, C12/IA-TP and C18/IA-TP, were blended at 10 and 25 wt % loading with MenMA and subsequently cured using UV light to produce thermosets denoted as C12/IA-meth and C18/IA-meth. PM resins containing 25 wt % C12-TP and C18-TP in MenMA were also formulated to compare the effects of thermoplastic polyesters with and without IA on the polymer properties. PM resins without IA content are denoted as C12-meth and C18-meth. Neat uncured MenMA and cured PMenMA were also evaluated for comparison. We observed an increase in liquid resin viscosity with an increased loading of the thermoplastic polyester precursor for both C12- and C18-based resins (Figure S30 and Table S3). All resins exhibited viscosities less than 1000 cP at 25 °C, which is adequate for resin-based 3D printing applications.^46^

**Figure 2:**
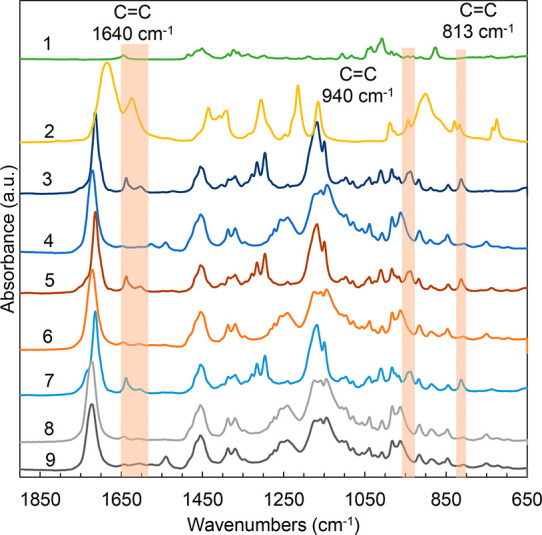
Representative FTIR spectra of (1) betulin, (2) IA, (3) MenMA monomer, (4) PMenMA, (5) 10 wt % C12/IA-meth uncured, (6) 10 wt % C12/IA-meth cured, (7) 25 wt % C12/IA-meth uncured, (8) 25 wt % C12/IA-meth cured, and (9) 25 wt % C12-meth cured.

The conversion of the C=C bonds from the PM resins upon UV curing was monitored by using FTIR spectroscopy. Peaks at 1640, 940, and 813 cm^–1^ (Figure 2 and Figure S34) were present for both IA and the uncured MenMA monomer, which correspond to the stretching and deformation vibrations from the C=C bonds.^32,45^ Comparing uncured resins to cured sequential thermosets, bands at 940 and 813 cm^–1^ disappeared after UV curing, indicating overall high conversion. Residual signals at 1640 cm^–1^ after curing could be due to residual unsaturations present; however, betulin exhibits a peak near 1640 cm^–1^ from the isopropenyl unsaturation.^47^ Because of the close proximity of peaks around 1640 cm^–1^, the signal at 3075 cm^–1^ was identified as the C–H alkene stretching from the betulin moiety (Figures S32 and S35). This peak was present in all uncured resins and cured samples containing thermoplastic polyester precursors, indicating that C=C bonds from betulin remained after curing. The peak absorbance of this band was most prominent for resins with 25 wt % thermoplastic polyester loading. Additionally, the disappearance of methacrylate peaks in the ^1^H NMR spectrum for PMenMA (Figure S21) compared to the uncured MenMA monomer (Figure S20) supports a high extent of curing of the methacrylate network at the chosen UV curing conditions.

**Figure 3:**
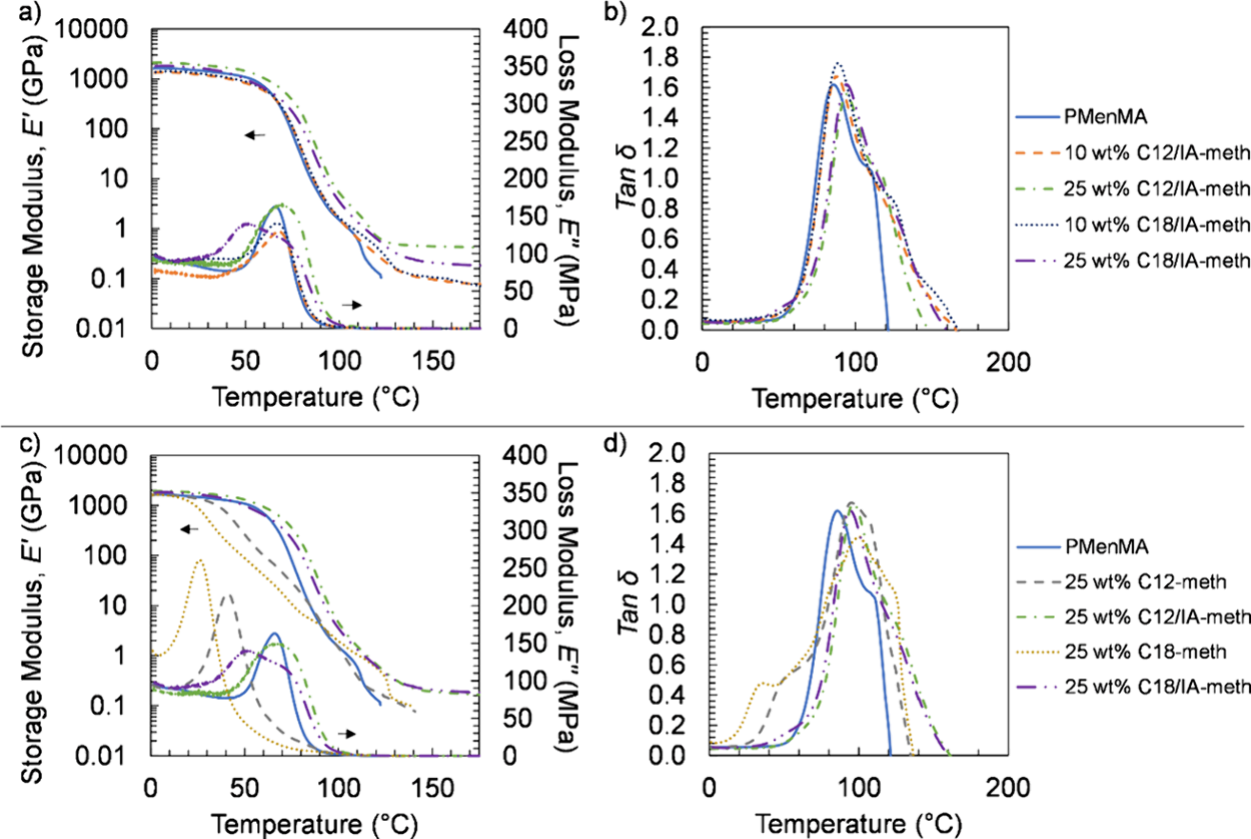
DMA comparing (a,b) representative *E′*, *E″*, and tan δ curves for PM thermosets with IA and (c,d) representative *E′*, *E″*, and tan δ curves for PM polymers with and without IA.

Thermomechanical properties of the cured PM resins were characterized by using dynamic mechanical analysis (DMA), with results shown in Table 2 and Figure 3. We aimed to transform thermoplastic polymethacrylates into cross-linked thermosets using IA-containing thermoplastic polyester precursors via photopolymerization. Since MenMA is a monomethacrylate and forms a fully thermoplastic network upon curing, PMenMA was characterized as a baseline to compare the properties of resins without added thermoplastic polyester precursors. PMenMA exhibited a sharp decrease in storage modulus around 115 °C. This decrease for amorphous thermoplastic materials is indicative of melting, where large-scale chain slippage occurs and material flows.^48^ Consequently, PMenMA yielded before the DMA test was able to reach completion at 175 °C. The storage moduli (*E′*) at 25 °C did not change upon adding 10 wt % C12/IA-meth or C18/IA-meth compared to PMenMA. However, there was an appreciable increase in *E′* at 25 °C with increased thermoplastic polyester content from 10 to 25 wt %, where the *E′* at 25 °C increased from 1454 to 1790 MPa and from 1337 to 1618 MPa for C12/IA-meth and C18/IA-meth cured resins, respectively.

**Table 2:** Viscoelastic Properties of Sequentially Prepared PM Polymers

**cured resin**	***E′*****at 25 °C (MPa)**	**peak of*****E″*****(°C)**	**peak oftan δ(°C)**	**rubbery*****E′*****(MPa)^a^**	**ν**_*e*_**(mol m^–3^)**
PMenMA	1335 ± 101	64 ± 2	84 ± 2		
10 wt % C12/IA-meth	1454 ± 243	70 ± 2	89 ± 1	0.15 ± 0.04	14 ± 4
25 wt % C12/IA-meth	1790 ± 69	69 ± 1	95 ± 1	0.26 ± 0.13	25 ± 12
25 wt % C12-meth	1473 ± 116	43 ± 2	48 ± 7/95 ± 5		
10 wt % C18/IA-meth	1337 ± 55	67 ± 1	87 ± 1	0.14 ± 0.03	14 ± 3
25 wt % C18/IA-meth	1618 ± 105	52 ± 4/72 ± 2	94 ± 1	0.29 ± 0.09	28 ± 8
25 wt % C18-meth	843 ± 57	27 ± 1	34 ± 1/100 ± 3		

a Rubbery *E′* at *T*_g,tan δ_ + 50 °C.

A rubbery plateau was observed for all sequentially prepared thermosets (Figure 3a). This plateau and elimination of a molten region indicated the formation of cross-linked networks.^49,50^ The cross-link densities, ν_*e*_, of cured resins with 25 wt % loading of IA thermoplastic polyester precursor were higher than those with 10 wt % loading, which may be due to increased IA content, although the averages for the C12/IA-meth cured resins fall within experimental error. The ν_*e*_ did not vary between C12/IA-meth and C18/IA-meth resins at all thermoplastic polyester precursor loadings, suggesting that the difference in the chain length of the diacids did not have an appreciable effect on ν_*e*_. Additionally, the gel content increased with increasing thermoplastic polyester precursor loading, and no gel content was observed for the thermoplastic PMenMA, corresponding to the increase in calculated ν_*e*_ (Table S4).

The peaks of the loss moduli (*E″*) did not vary for the sequentially prepared thermosets compared to PMenMA. Both the 25 wt % C12/IA-meth and C18/IA-meth cured resins exhibited broader *E*″ curves. This broadening can be attributed to an increase in heterogeneity of the polymers.^51^ More branched side products could have been incorporated with a higher thermoplastic polyester precursor loading. This may explain the increased opacity of samples due to phase separation and residual undissolved thermoplastic polyester precursor, especially for 25 wt % C18/IA-meth, which exhibited two distinct *E″* peaks.

A sharp drop was observed from the tan δ of PMenMA at 115 °C, which aligned with the melting and yielding of the polymer. Polymer backbones lose mobility with increased cross-linking, which has little effect on the glassy *E′* relative to effects on *E″* and tan δ, and typically, the loss of backbone mobility shifts *T*_g_ higher and broadens *E″* and tan δ peaks.^49^ This broadening was exhibited by all cured PM resins containing IA when compared to PMenMA. Additionally, higher *T*_g_ values were observed for the cured PM resins with a 25 wt % loading compared to 10 wt %, which can be attributed to higher cross-link densities. This increase in *T*_g_ was also observed by DSC (Table S4).

The thermomechanical properties of PM polymers without IA exhibited lower *E′* values compared to PM resins with IA. Like the thermoplastic PMenMA, these polymers underwent melting and were unable to produce a rubbery plateau due to the absence of cross-linking sites. These results were as expected as thermoplastic polyesters are known to be incompatible when blended with other thermoplastics such as polystyrene, polyolefins, and PMMA and require chemical modifications and compatibilizers to improve miscibility, interfacial adhesion, and resulting mechanical properties.^52^ The peak of *E″* values were lower for these PM resins without IA compared to those with IA, and the peak of the *E″* values were higher for the C12-based PM resin compared to C18 due to the shorter chain length resulting in higher *T*_g_. Two tan δ peaks indicated network heterogeneity, which similar studies have reported for phase-separated polymers with clusters of rigid and flexible domains.^53^ The lower temperature tan δ peak corresponds to the relaxation of a polyester-rich phase by the more flexible C12 and C18 alkyl chains. The higher temperature tan δ peak aligns with the more rigid PMenMA-rich phase and thus agrees with the tan δ peaks from the PMenMA homopolymer and PM resins containing IA.

These results demonstrate that a controlled synthesis of thermoplastic polyester precursors allows for modulation of precursor properties, and sequentially cured thermosets can be prepared with a varied precursor loading to tune the final thermomechanical properties. Sequentially prepared thermosets were cross-linked via incorporation of IA, avoiding direct (meth)acrylation of the betulin monomer, which may improve the life cycle impacts during production. While these materials may be suitable for applications requiring UV curing capabilities and low cross-link densities, these cured PM materials exhibited IDTs around 200 °C (Table S5), and other applications may require higher cross-link densities and better thermal stability. Furthermore, though thermoplastic polyester precursors enabled tunable properties, solvents were required for thermoplastic purification during synthesis. Therefore, to broaden the scope of use for thermosets produced from betulin and IA, we synthesized polyester thermosets using a solventless bulk curing procedure that used glycerol as a cross-linker.

### Polyester Thermosets Prepared in Bulk with Glycerol

#### Bulk-Cured Polyester Thermoset Synthesis and Properties

To expand the utility of betulin-based thermosets, a one-pot melt polycondensation procedure was performed using equimolar ratios of alcohol and carboxylic acid functional groups. Here, IA was incorporated into betulin-based thermosets formulated with long-chain diacids (C12 or C18) to evaluate its effect on the final thermoset properties. Similar to the sequentially prepared thermosets, a 50/50 molar ratio of the linear aliphatic diacid (C12 or C18) to IA was used but without a methacrylate monomer. Polyester thermosets with IA are denoted as C12/IA-glyc and C18/IA-glyc, and polyester thermosets without IA are denoted as C12-glyc and C18-glyc.

**Figure 4:**
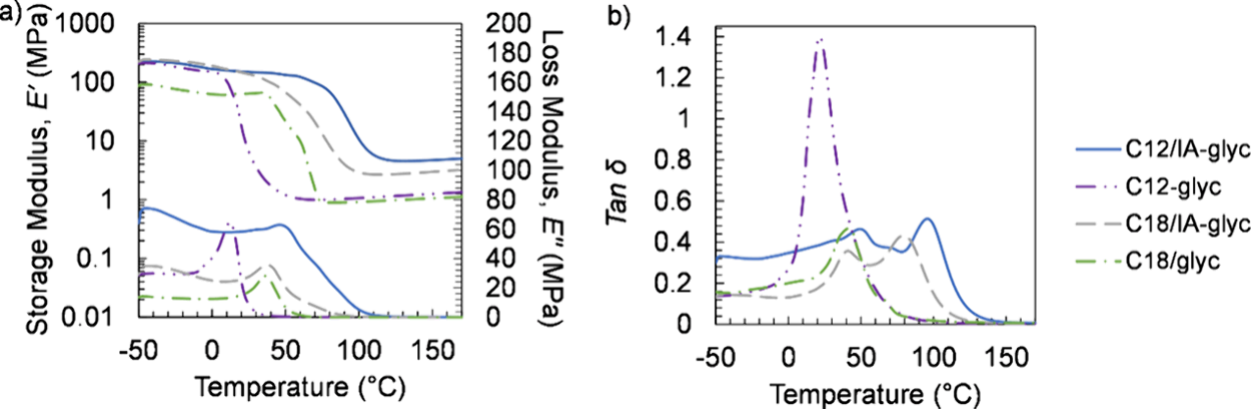
DMA comparing representative (a) *E′*, *E″*, and (b) tan δ curves for the bulk-cured polyester thermosets.

Thermomechanical properties of these polyester thermosets were characterized by using DMA (Figure 4 and Table 3). Polyester thermosets with 50/50 IA/diacid resulted in networks displaying a nearly 4 times greater *v*_*e*_ compared to their counterparts formed without IA for both C12- and C18-based polyester thermosets. Incorporating IA imparted shorter chain segments between cross-links, which increased the rubbery plateau *E′* and the *v*_*e*_ of the resulting polyester thermosets. Comparing C12- to C18-based polyester thermosets, decreasing the diacid chain length may have led to a small rise in the *v*_*e*_ in samples prepared with and without IA, though these estimates fall within experimental error. These studies also suggest that incorporating IA elevates *E*′ in the glassy region, which is attributed to a decrease in molecular weight between cross-links due to incorporation of IA in the polymer backbone.^54,55^ Samples with IA both exhibited two *T*_g_s as determined by the peaks of tan δ, likely owing to heterogeneity in the networks due to the presence of two diacids, similar to the previously discussed PM samples. Additionally, the second peaks of tan δ are elevated in networks containing IA compared with their counterparts formulated without IA.

**Table 3:** Viscoelastic Properties of Bulk-Cured Polyester Thermosets

**bulk-cured polyester thermoset**	***E′* at25** **°C(MPa)**	**peak of *E*″ (°C)**	**peak oftan δ(°C)**	**rubbery*****E′*****(MPa)^a^**	**ν_*e*_(mol m^–3^)**
C12-glyc	7 ± <1	12 ± 1	22 ± <1	0.99 ± 0.04	113 ± 3
C12/IA-glyc	162 ± 22	48 ± 3	51 ± 2/94 ± 2	4.16 ± 0.46	403 ± 45
C18-glyc	69 ± 5	36 ± <1	42 ± <1	0.82 ± 0.08	90 ± 9
C18/IA-glyc	131 ± 17	39 ± 3	43 ± 2/80 ± 1	3.70 ± 0.67	368 ± 66

a Rubbery *E′* at *T*_g,tan δ_ + 50 °C.

We note that when C18 is used as the only diacid in the thermoset network, semi-crystalline polyesters are formed as observed by a clear melting peak at 56 °C from DSC (Figure S42). C18-glyc polyester thermosets exhibited elevated *T*_g_s (41.7 ± 0.1 °C from the tan δ peak) compared to C12-glyc polyester thermosets (22.8 ± 0.4 °C) likely due to the packing of the longer carbon chains in the C18-glyc samples. Moreover, the tan δ peak height for the C12-glyc polyester thermoset was the greatest, suggesting that the C12-glyc polyester thermosets show enhanced energy dissipation compared with the C18-glyc polyester thermosets, likely due to the presence of crystalline regions in the C18-glyc polyester thermosets.

**Figure 5:**
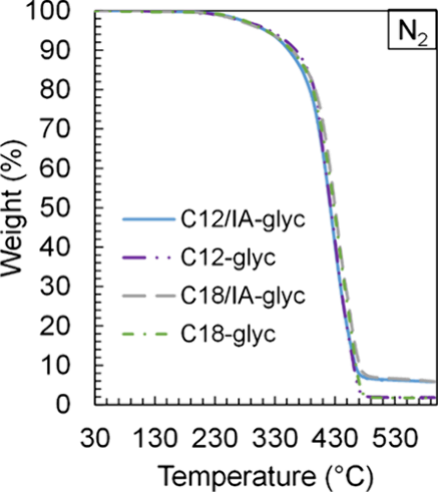
Representative TGA thermograms of the bulk-cured polyester thermosets in N_2_.

All polyester thermosets exhibited excellent thermal stability in both inert (N_2_) and oxidative (air) environments, with IDTs and *T*_50_s occurring above 295 and 420 °C, respectively. No polymers show appreciable mass loss near 100 °C, indicating that the water byproduct was removed during curing. As shown in Figure 5 and Table 4, the addition of IA as a comonomer may modestly increase the *T*_50_ value in both N_2_ and air except for polyester thermosets prepared with C12 and tested in N_2_ where the *T*_50_ remains constant. Moreover, networks containing IA showed less thermal degradation at temperatures around 475 °C, resulting in higher char yield values. These higher char yields are likely due to the increased cross-link densities of those polyester thermosets. As expected, the degradation attributable to C–C bond cleavage is more apparent in the oxidative environment. We also find that increasing the diacid chain length from C12 to C18 raises the *T*_50_s in samples prepared with and without IA when tested in both N_2_ and air, likely due to the lower molecular weight fraction of ester groups per repeat unit in polymers formulated with the longer-chain diacid. Thus, these results support the notion that betulin-based polyester thermosets formulated with long- (C12 and C18) and short-chain (IA) diacids can be used in high-temperature applications in both oxidative and inert environments.

**Table 4:** Thermogravimetric Results for the Bulk-Cured Polyester Thermosets

**bulk-cured polyester thermoset**	**N**_**2**_			**air**		
	**IDT (°C)**	*T***_50_(°C)**	**char yield (%)**	**IDT (°C)**	*T***_50_(°C)**	**char yield (%)^a^**
C12/IA-glyc	309 ± 5	423 ± 1	5.42 ± 0.44	298 ± 4	434 ± 1	0.64 ± 0.03
C12-glyc	317 ± 5	424 ± <1	3.70 ± 1.88	307 ± 3	431 ± <1	0.48 ± 0.05
C18/IA-glyc	318 ± 3	430 ± <1	4.94 ± 0.57	314 ± 3	441 ± 1	1.07 ± 0.23
C18-glyc	315 ± <1	427 ± 2	1.79 ± 0.05	311 ± 1	437 ± <1	0.80 ± 0.09

a Char yield values determined at 600 °C.

These findings demonstrate that IA can be incorporated as a comonomer into betulin-based polyester thermosets prepared in bulk for tuning final thermoset properties. The properties of these thermosets could also be further tuned by varying the ratios of the initial monomers used. Controlled cross-linking of IA for these bulk-cured polyester thermosets was not discussed in this work but will be the subject of future studies. Compared with the PM thermosets, these fully polyester thermosets possessed higher fractions of ester bonds, producing qualitatively tougher materials. The bulk-cured polyester thermosets also demonstrated higher thermal stabilities and higher ν_*e*_s compared to the sequentially prepared PM thermosets, though these properties should be weighed against their lower *E′* and lower *T*_g_ values when considering appropriate applications of these materials.

## Conclusions

Itaconic acid (IA) and betulin were successfully utilized as biobased monomers to prepare thermosets using sequential and bulk curing methods. These different approaches highlight the versatility of IA as a comonomer to produce both UV-curable and thermally curable betulin-based thermosets. We first synthesized thermoplastic polyester precursors to sequentially prepare PM thermosets, demonstrating that thermoplastics containing IA were able to photopolymerize with a biobased monomethacrylate monomer. The sequentially prepared thermoset properties were tuned as ν_*e*_, gel content, and *E*′ at 25 °C all increased with higher weight percent loadings of the thermoplastic polyester precursor. When incorporating IA as a shorter-chain diacid comonomer in bulk-cured glycerol-based thermosets, we observed enhancement of thermomechanical properties compared to thermosets without IA while maintaining high thermal stabilities of approximately 300 °C.

Both curing methods provided distinctive processing and property advantages with different potential life cycle impacts on manufacturing and end-of-life phases, which could affect the suitability of these thermosets for chosen applications. The sequentially prepared thermosets possessed high levels of tunability from the controlled syntheses of the thermoplastic precursors, and these polyester-methacrylate (PM) resins were able to undergo UV curing at relatively low temperatures without the need for a multistep (meth)acrylation of betulin. Using IA to prepare unsaturated betulin-based precursors presents an alternative method for incorporating betulin in UV-curable applications, such as coatings, biomedical applications, and 3D printing resins. Alternatively, the bulk-cured thermosets were cured using a one-pot thermal synthesis, and although the cross-linking of these glycerol-based thermosets could not be spatially controlled, this bulk approach allowed for a solventless thermoset preparation without any prior monomer modifications. Higher synthesis temperatures were used compared to the UV-cured PM resins, but the final materials ultimately possessed higher cross-link densities and better thermal stabilities. Moreover, these thermally cured thermosets consisted fully of ester linkages with consequently different degradation capabilities compared to the PM thermosets, which have promising implications for the potential end-of-life impacts of these materials. Studies are currently underway to better understand the hydrolytic stability and degradability of these fully polyester thermosets.

Overall, this work highlights two viable methods for using IA as a comonomer to form betulin-based thermosets with tunable properties. IA and betulin were strategically chosen as biocompatible monomers to potentially mitigate the impacts of these materials during the use phase of the life cycle. The potential life cycle impacts regarding the manufacturing and end-of-life of these thermosets were discussed. Both sequentially prepared and bulk-prepared thermosets have potential to contain 100% biobased carbon content; C12, C18, and glycerol can be derived from plant-sourced oil feedstocks, and methacrylic acid has the potential to be produced from fermentation and enzymatic pathways.^31,56^ Both UV and thermally curable thermosets presented have the potential to be used in a wide array of applications depending on desired processing and properties, widening the scope for the incorporation of betulin and IA in polymeric materials and further enhancing sustainable manufacturing.

## Supporting Information Available

Additional synthesis and characterization details; ^1^H NMR and ^13^C NMR spectra; BDMI characterizations; additional APC traces, DSC results, and TGA thermograms for thermoplastic polyester precursors; rheology results for PM resins; appearance of cured PM resins; additional FTIR spectra; DSC and gel contents of cured PM resins; TGA results of cured PM resins; DSC and additional TGA results for bulk-cured polyester thermosets (PDF)
