# Supplementary material for: Itaconic Acid as a Comonomer in Betulin-Based Thermosets via Sequential and Bulk Preparation
Source: ACS Sustain Chem Eng. 2023 Sep 13;11(38):14216–25. doi: 10.1021/acssuschemeng.3c04178 (PMC10526528; doi:10.1021/acssuschemeng.3c04178)
Supplement: Supplementary file 1 — sc3c04178_si_001.pdf [file sc3c04178_si_001.pdf]

SUPPORTING INFORMATION

Itaconic Acid as a Co-monomer in Betulin-based Thermosets via Sequential and Bulk Preparation

Alexandra M. Lehman-Chong,[ab] Casey L. Cox,[ab] Emre Kinaci,[b] Sarah E. Burkert,[c] Megan L. Dodge,[c] Devin M. Rosmarin,[c] James A. Newell,[ab] Lindsay Soh,[c] Melissa B. Gordon,[c] Joseph F. Stanzione, III[ab*]

[a]*Department of Chemical Engineering, Rowan University, 201 Mullica Hill Road, Glassboro, New Jersey 08028, United States*
[b]*Advanced Materials & Manufacturing Institute (AMMI), Rowan University, 201 Mullica Hill Road, Glassboro, New Jersey 08028, United States*
[c]*Department of Chemical and Biomolecular Engineering, Lafayette College, 740 High Street, Easton, Pennsylvania 18042, United States*

*Corresponding Author: stanzione@rowan.edu

This supplemental document contains 29 pages (Figures S1-S44, and Tables S1-S5).

Contents

1. Synthesis Details for Thermoplastic Precursors
2. Synthesis Details for Menthyl Methacrylate (MenMA)
3. Characterization Methods
4. Thermoplastic Polyester Yields
5. ^{1}H-NMR and ^{13}C-NMR Spectra
6. BDMI Characterizations
7. Additional APC Traces for Thermoplastic Precursors
8. Additional DSC Traces for Thermoplastic Precursors
9. TGA Results for Thermoplastic Precursors
10. Rheology Results for PM Resins
11. Appearance of Cured PM Resins
12. Additional FTIR Spectra for Cured and Uncured PM Resins and Thermoplastic Precursors
13. DSC and Gel Content Results for PM Cured Resins
14. TGA Results for PM Cured Resins and Cured PMenMA
15. DSC Results for Bulk-cured Polyester Thermosets
16. Additional TGA Results for Bulk-cured Polyester Thermosets

1. **Synthesis Details for Thermoplastic Precursors**

Betulin (4.52 mmol, 2 g) and the chosen diacid(s) were added to a two-neck 25 mL round bottom flask in a 1:1 molar ratio of betulin to total diacid, accounting for reagent purity. DBTO catalyst (0.6 mol%, 6.7 mg) and a magnetic stirrer were added, and the flask was placed in a metal Heidolph heating mantle covered with aluminum foil. The flask was equipped with an inline cold trap connected to a condenser, and an external cold trap was connected to the vacuum pump.[1] The reagents were stirred at 50 rpm for 15 min at room temperature while purging with argon. Then the mixture was heated to 180 °C and left to react for 3 h. The argon line was then replaced with a vacuum line set to 500 mbar and left for 2 h. Finally, the pressure was further decreased to 0.8 mbar and left for 43 h. The resulting product was dissolved in DCM, reprecipitated in cold methanol, and left in a 40 °C vacuum oven until the product was determined to be dry according to ^{1}H-NMR spectroscopy.

The synthesis of a homopolymer using only betulin and IA was attempted. However, these monomers did not undergo adequate melting and resulted in poor mixing at the chosen reaction conditions. Alternatively, dimethyl itaconate (DMI) was used to synthesize a thermoplastic homopolymer, poly(betulin-dimethyl itaconate) (BDMI). A 1:1 molar ratio of betulin to DMI was used following the same procedure described above.

2. **Synthesis Details for Menthyl Methacrylate (MenMA)**

Menthol (1 mol, 156 g), methacrylic anhydride (1.1 mol, 10% excess per -OH, 185 g), and 4-DMAP (1 wt%, 3.4 g) were mixed in a four-neck round bottom flask equipped with a mechanical stirrer, argon inlet, temperature probe, and condenser. Contents were mixed under reflux at 65 ºC for at least 12 hours with continuous argon purge. The reaction progress was monitored via acid number titrations according to ASTM D664. Contents were then cooled to ambient temperature and mixed with 250 mL ethyl acetate. The solution was washed with 15% NaOH (aq) (200 mL × 3), DI water (100 mL × 1), and 15% brine (200 mL × 3) to remove excess methacrylic anhydride and methacrylic acid formed as a side product. The organics were dried on $MgSO_4$ overnight, filtered, and rotary evaporated to obtain a clear liquid. 1000 ppm hydroquinone was added to the final product (yield: ~80%).

3. **Characterization Methods**

Nuclear Magnetic Resonance Spectroscopy

Polyester thermoplastic precursors, MenMA resin, and cured PMenMA were characterized by ^{1}H-NMR spectroscopy (16 scans, 298 K) using a Bruker AdvanceCore Nuclear Magnetic Resonance (NMR) Spectrometer equipped with an Ascend 400 magnet. Thermoplastic precursors were also characterized by ^{13}C-NMR spectroscopy (1024 scans, 298 K). Approximately 25 mg of sample was dissolved in 1 mL of $CDCl_3$ for all samples.

Advanced Polymer Chromatography

The number average molecular weight (M_n), weight average molecular weight (M_w), and dispersity, M_w/M_n ($Đ$) of the thermoplastics were characterized using a Waters ACQUITY Advanced Polymer Chromatography (APC) system calibrated with polystyrene standards (PSS

ReadyCal Kit, range: 474 – 2500000 g mol^{-1}; maximum *Đ*: 1.15). Samples were prepared in THF at a concentration of 10 mg mL^{-1}, and THF was used as the eluent at a flow rate of 0.6 mL min^{-1}.

Differential Scanning Calorimetry

Differential scanning calorimetry (DSC) was performed using a TA Instruments Discovery Series DSC 2500. 5-10 mg samples were hermetically sealed in Tzero aluminum pans. All samples were equilibrated at 0 °C, heated at a rate of 10 °C min^{-1}, cooled at a rate of 40 °C min^{-1}, and heated again at 10 °C min^{-1}. Thermoplastic precursors were heated to 200 °C and cooled to 0 °C. Cured PM resins were heated to 175 °C and cooled to 0 °C. Bulk-cured thermosets were heated to 250 °C and cooled to -25 °C. Experiments were performed under N_2 flow at 50 mL min^{-1}. The glass transition temperatures (T_gs) were determined by the second heating ramp, and any applicable melting temperatures (T_ms) were determined.

Rheology

Uncured PM resin viscosities were determined using a TA Instruments Discovery Hybrid Rheometer (Discovery HR-2). A 1° 40 mm cone geometry with the Peltier plate was conditioned at 25 °C with an equilibration time of 120 seconds. The shear rate was increased logarithmically from 1 s^{-1} to 100 s^{-1} at 5 points per decade and then decreased back to a shear rate of 1 s^{-1} at 3 points per decade.

Fourier Transform Infrared Spectroscopy

Attenuated total reflectance Fourier transform infrared (ATR-FTIR) spectroscopy was used to monitor the extent of cure of the PM resins with a Thermo Scientific Nicolet iS50 FTIR spectrometer. FTIR spectra (32 scans, 2 cm^{-1} resolution) were obtained at room temperature and obtained before and after curing in the range of 650 − 4000 cm^{-1}.

Gel Content

The gel content of the cured PM resins was determined. A solid sample (~0.5 g) was weighed and wrapped in two sheets of filter paper to form a parcel, and the initial weight was recorded. The specimen was continuously extracted for 24 hours with a Soxhlet apparatus using DCM and then dried in a vacuum oven at room temperature. The final gel content was calculated using the following equation,

$$Gel\ content = \frac{W}{W_0} * 100 \tag{1}$$

where W is the mass of sample after extraction and W_0 is the initial mass.

Thermogravimetric Analysis

Thermogravimetric properties were measured using a TA Instruments Discovery Series TGA 550. Thermoplastic precursors and cured PM resins (~10 mg) were placed on a platinum TGA pan and heated at a rate of 10 °C min^{-1} from 30 °C to 700 °C under both N_2 and air (25 mL min^{-1} sample gas flowrate). Bulk-cured specimens (6-9 mg) were heated from room temperature to 600 °C at 10 °C min^{-1} under both N_2 and air (60 mL min^{-1} sample gas flowrate). A balance gas flowrate of 40 mL min^{-1} was used for all experiments. The initial decomposition temperature (IDT) was defined at 5% weight loss. The temperature at 50% weight loss (T_{50}),

temperature at maximum degradation rate (T_{max}), and residual percent char content were also determined.

Dynamic Mechanical Analysis

The viscoelastic behavior of the sequentially-prepared PM resins and bulk-cured thermosets were measured using a TA Instruments Q800 Dynamic Mechanical Analyzer (DMA). Cured PM resins were cut and sanded to 35 mm x 12 mm x 3.0 mm dimensions. Samples were loaded on a single cantilever fixture with 2.5 in-lb of torque and heated at a rate of 2 °C min^{-1} from 0 °C to 175 °C. An oscillating frequency of 1 Hz, amplitude of 5.0 μm, and Poisson's ratio of 0.35 were used. Bulk-cured samples (22.25 mm × 8 mm × 1.7 mm) were measured using a 0.10% strain amplitude at 1 Hz in a film tension clamp with a preload force of 0.1 N. Temperature sweeps were performed using a heat-cool-heat method using a heating ramp rate of 3 °C min^{-1} and a cooling rate of 10 °C min^{-1} from -50 °C to 170 °C, where data was collected upon the second heating ramp. The storage modulus at 25 °C (E') and T_g, represented by the peak of the loss modulus (E'') and peak of the *tan δ* curves, were determined. The crosslink density (ν_e) was calculated according to an expression derived from the theory of rubbery elasticity,

$$\nu_e = \frac{E'_R}{3RT} \tag{2}$$

where E_R' is the rubbery storage modulus at $T_{g,tan\delta}$ + 50 °C, R is the ideal gas constant, and T is the absolute temperature corresponding to E_R'.[31, 38]

4. Thermoplastic Polyester Yields

Table S1. Thermoplastic polyester yields

Diacid	Diacid/IA Charged in Reaction (mol/mol)	Yield (%)	Thermoplastic Polyester
C12	100/0	86 ± 12	C12-TP
C12	25/75	90 ± 8	C12/IA75-TP
C12	50/50	79 ± 4	C12/IA-TP
C12	75/25	50 ± 12	C12/IA25-TP
C18	100/0	75 ± 3	C18-TP
C18	25/75	79 ± 8	C18/IA25-TP
C18	50/50	77 ± 6	C18/IA-TP
C18	75/25	60 ± 17	C18/IA75-TP
DMI	0/100	16 ± 3	BDMI

5. ^{1}H-NMR and ^{13}C-NMR Spectra

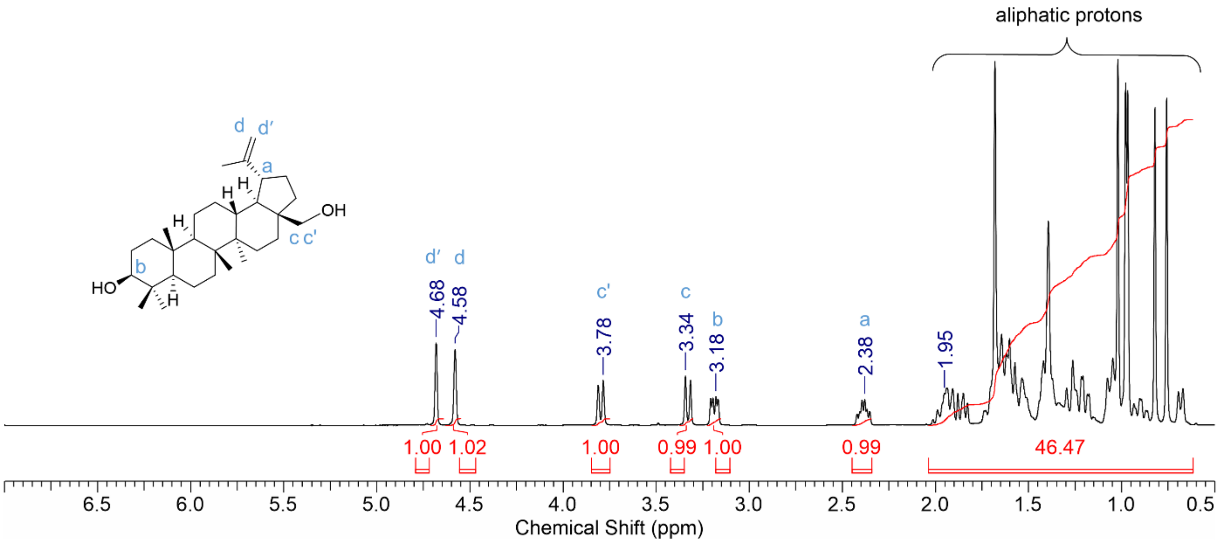

Figure S1. ^{1}H-NMR spectrum for betulin

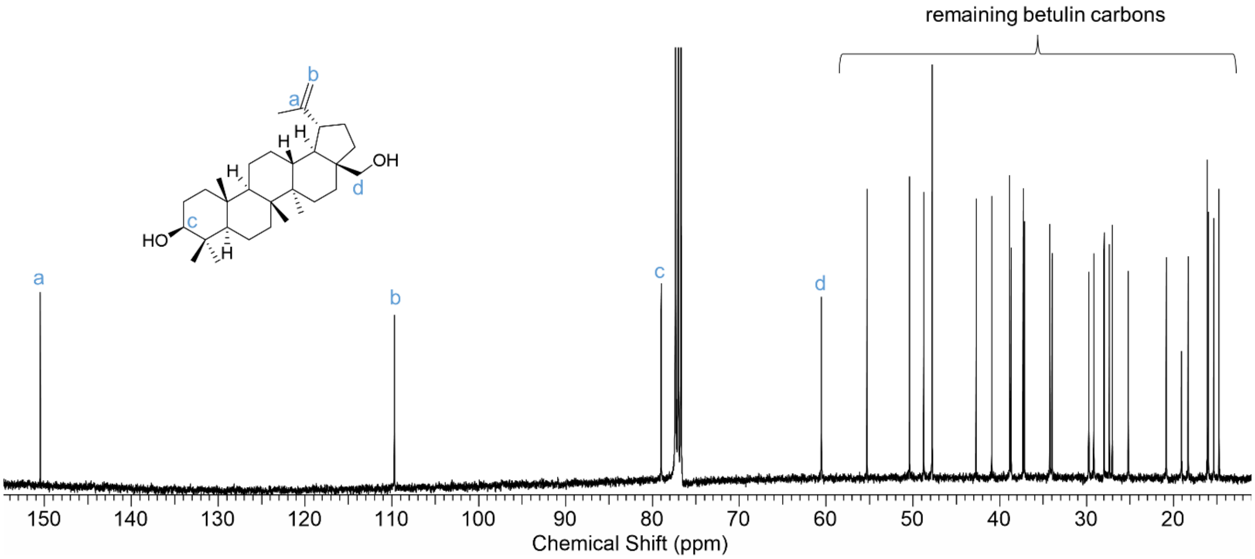

Figure S2. ^{13}C-NMR spectrum for betulin

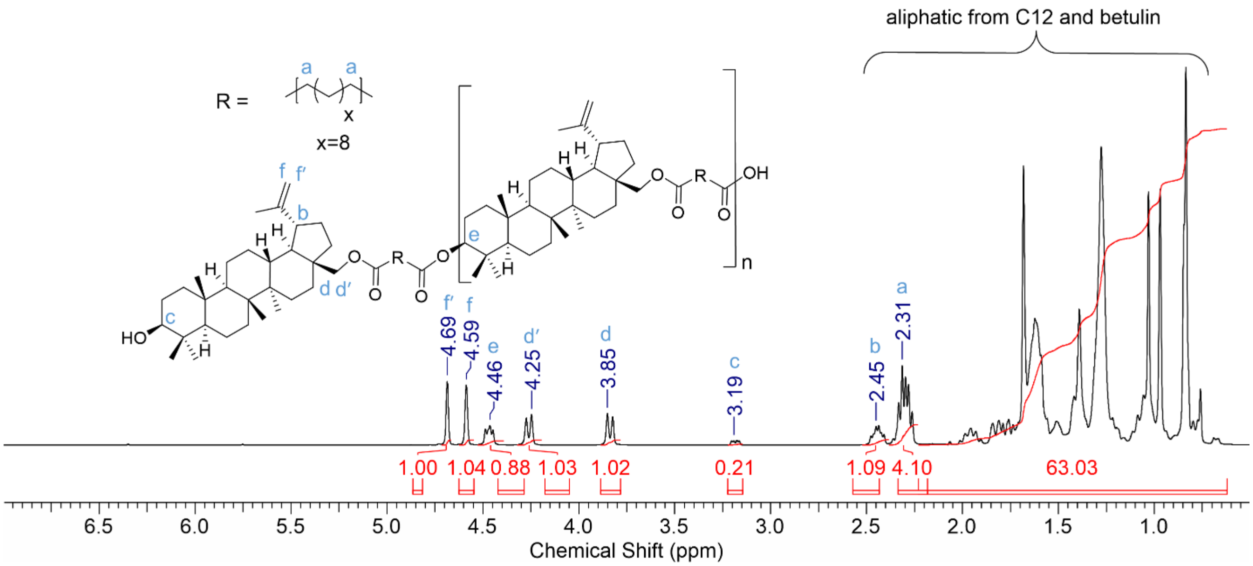

Figure S3. Representative ^{1}H-NMR spectrum for C12-TP

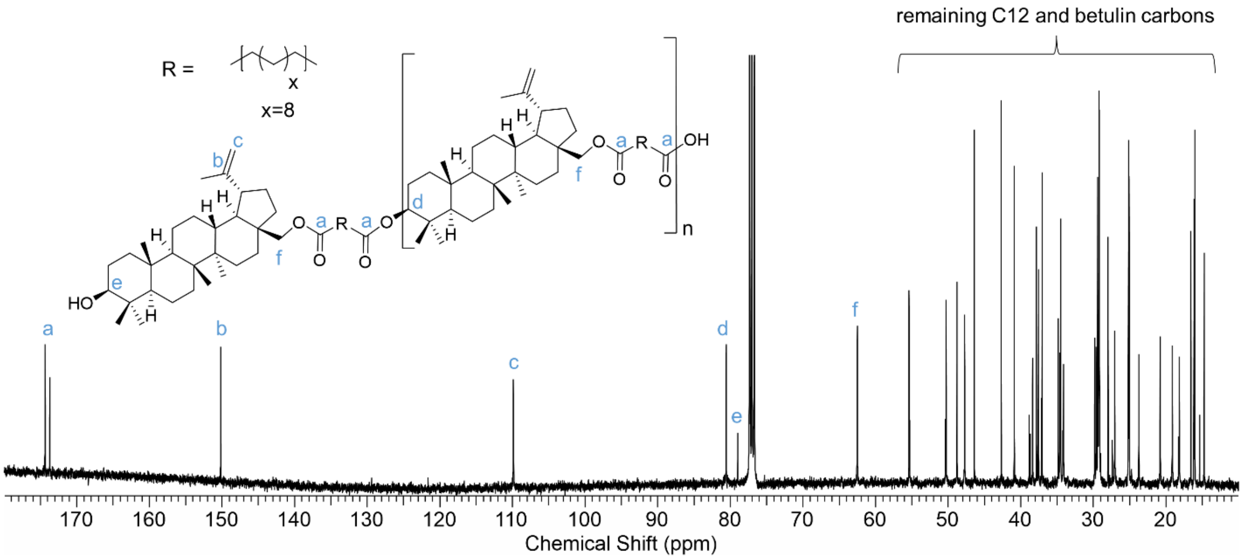

Figure S4. Representative ^{13}C-NMR spectrum for C12-TP

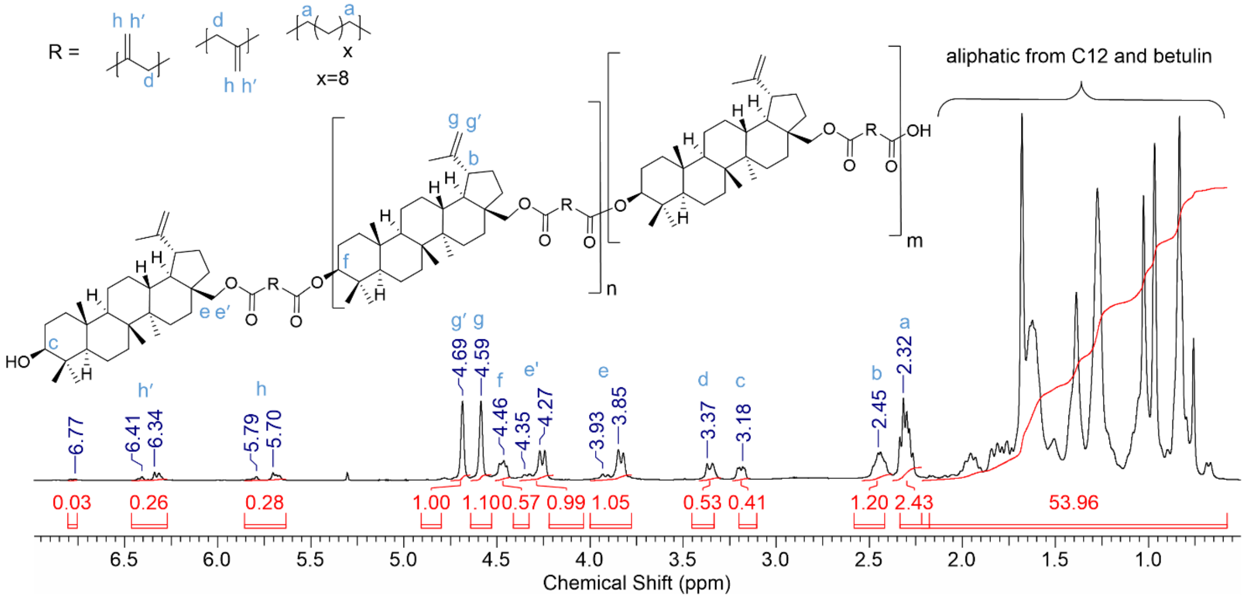

Figure S5. Representative ^{1}H-NMR spectrum for C12/IA-TP

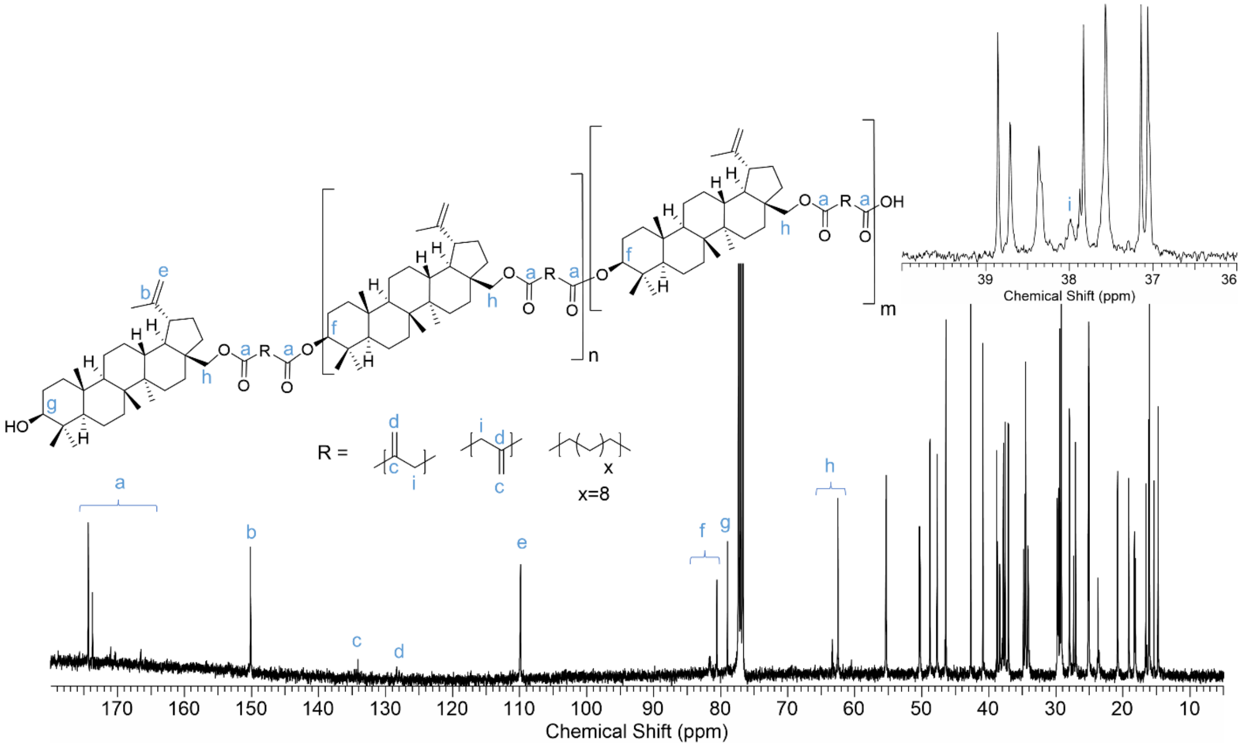

Figure S6. Representative ^{13}C-NMR spectrum for C12/IA-TP

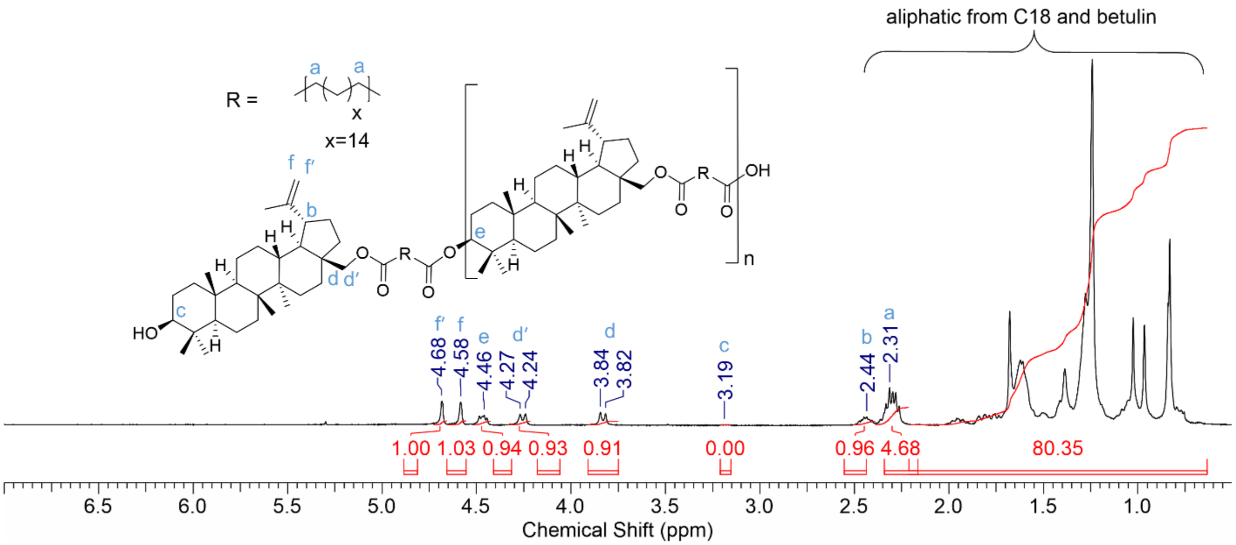

Figure S7. Representative ^{1}H-NMR spectrum for C18-TP

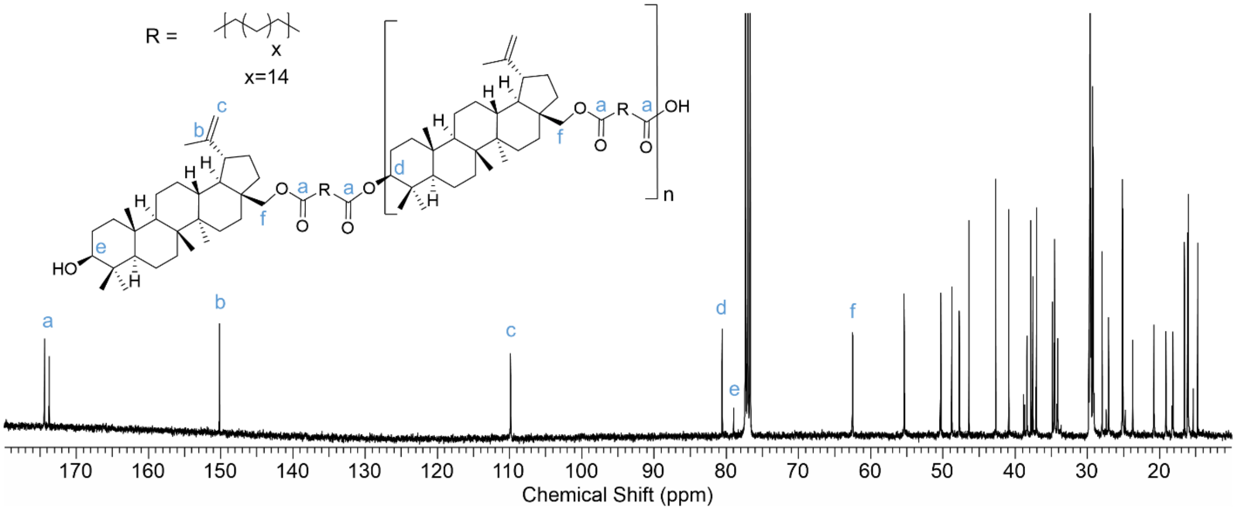

Figure S8. Representative ^{13}C-NMR spectrum for C18-TP

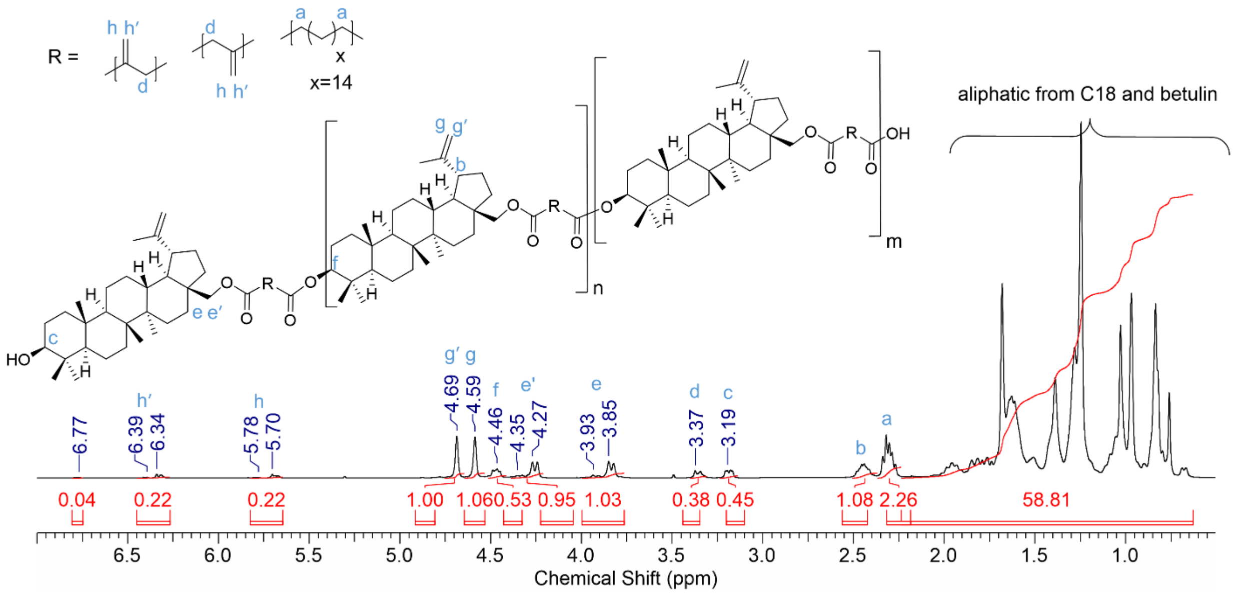

Figure S9. Representative ^{1}H-NMR spectrum for C18/IA-TP

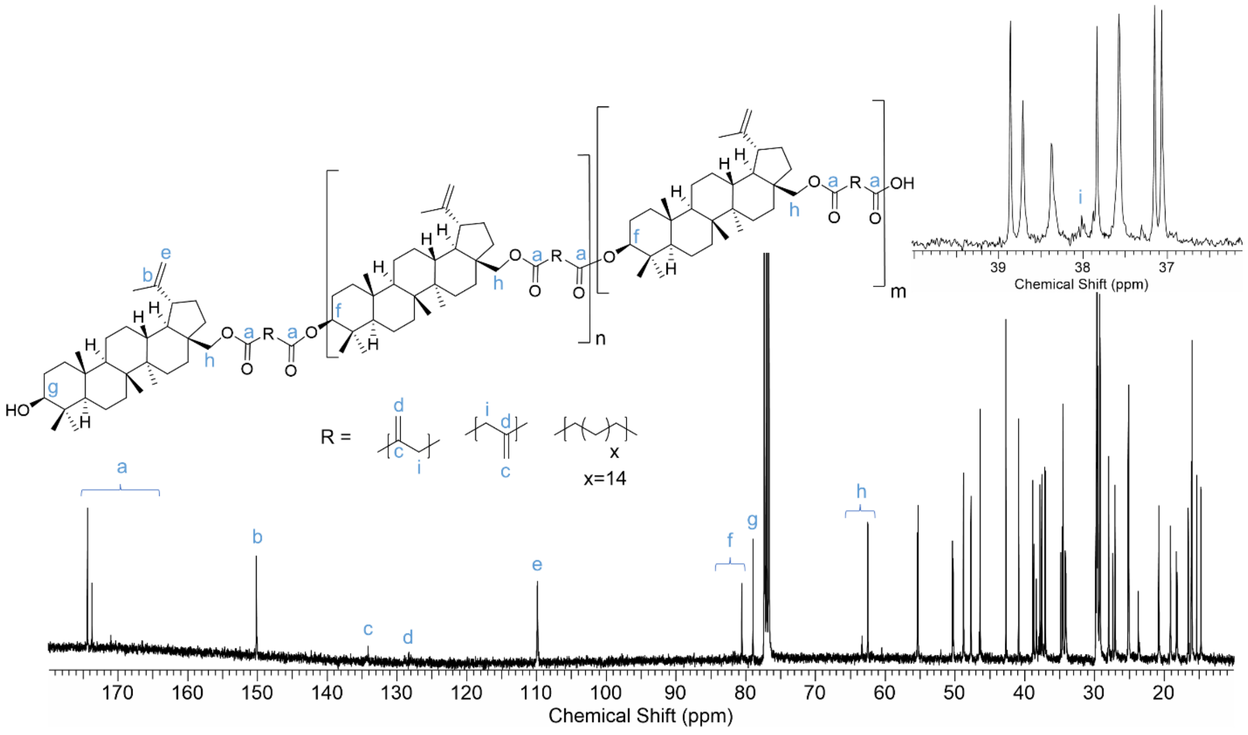

Figure S10. Representative ^{13}C-NMR spectrum for C18/IA-TP

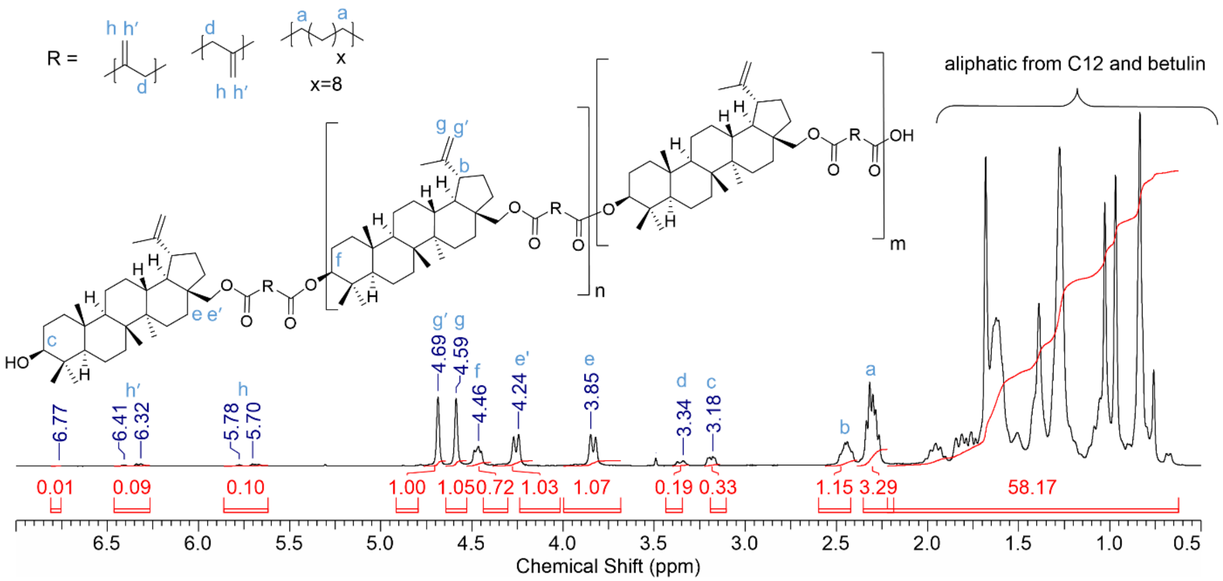

Figure S11. Representative ^{1}H-NMR spectrum for C12/IA25-TP

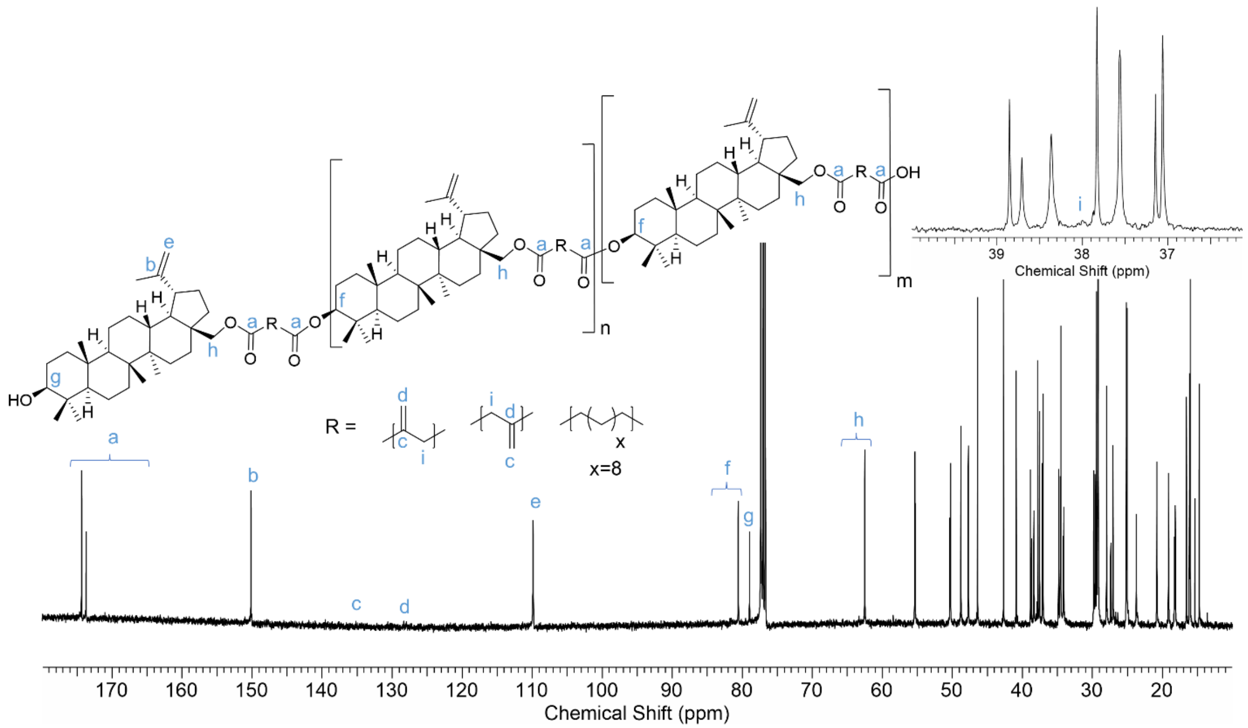

Figure S12. Representative ^{13}C-NMR spectrum for C12/IA25-TP

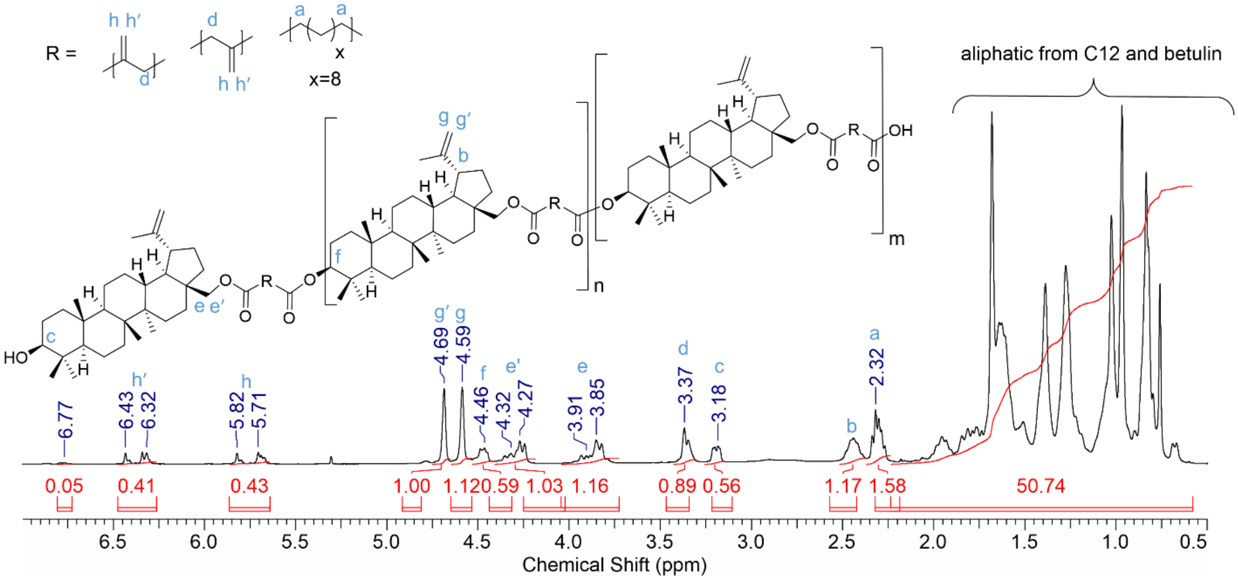

Figure S13. Representative ^{1}H-NMR spectrum for C12/IA75-TP

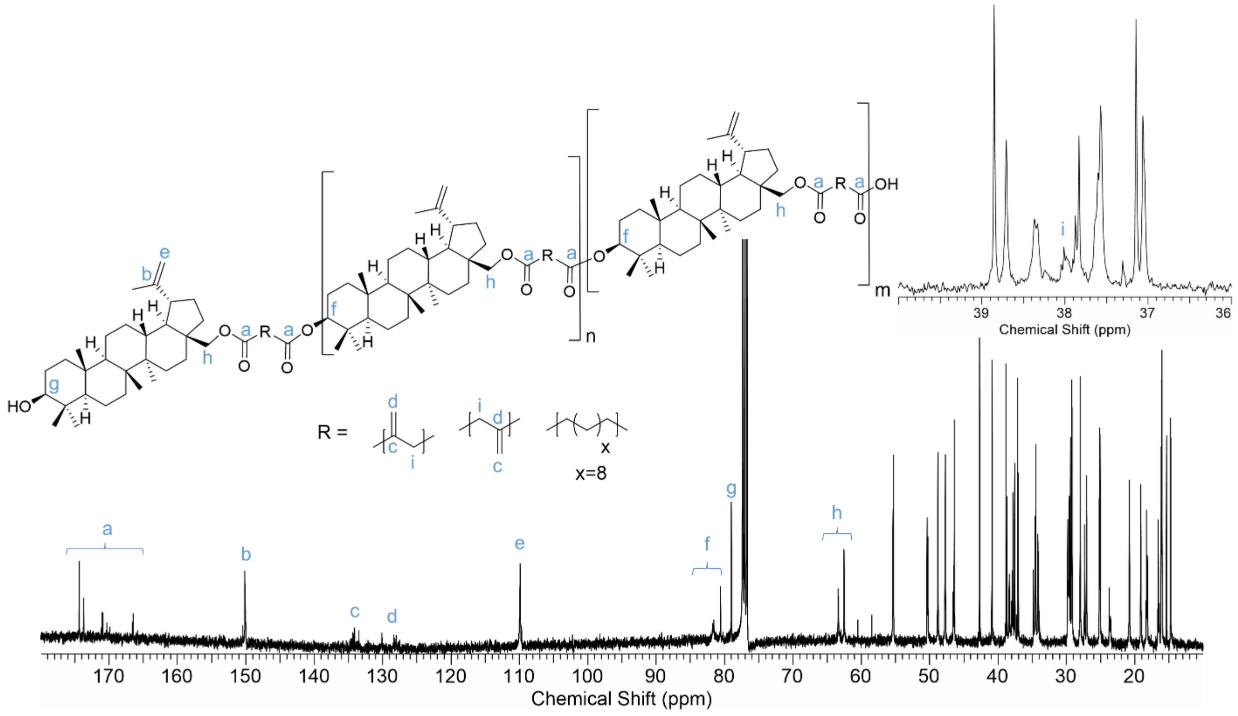

Figure S14. Representative ^{13}C-NMR spectrum for C12/IA75-TP

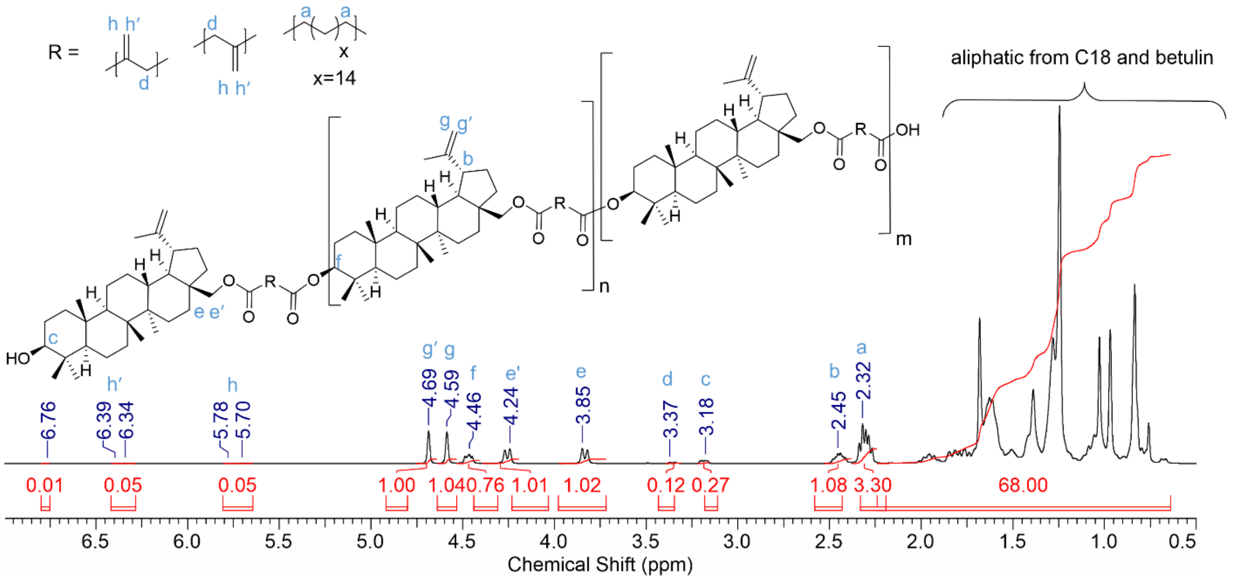

Figure S15. Representative ^{1}H-NMR spectrum for C18/IA25-TP

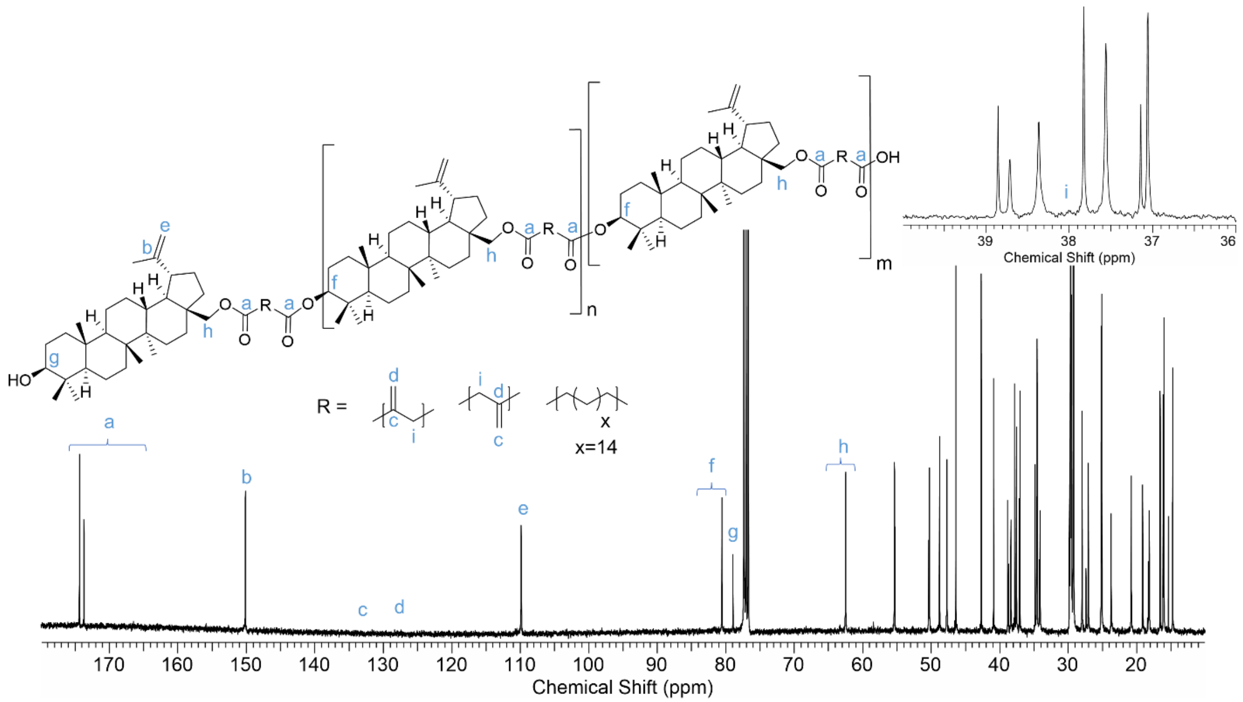

Figure S16. Representative ^{13}C-NMR spectrum for C18/IA25-TP

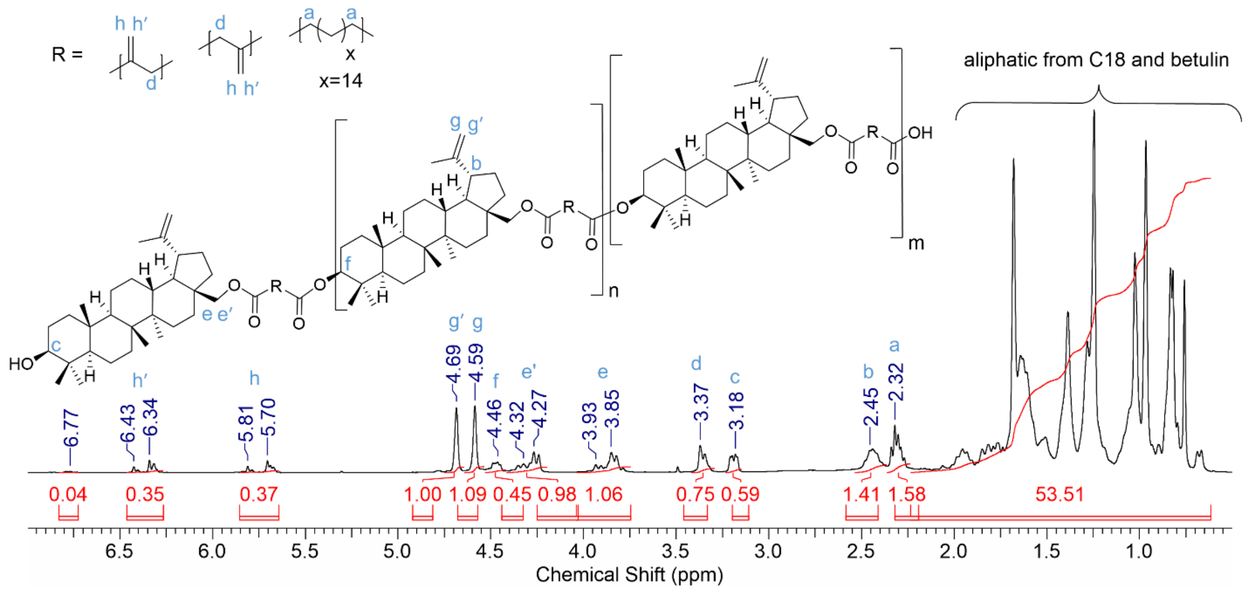

Figure S17. Representative ^{1}H-NMR spectrum for C18/IA75-TP

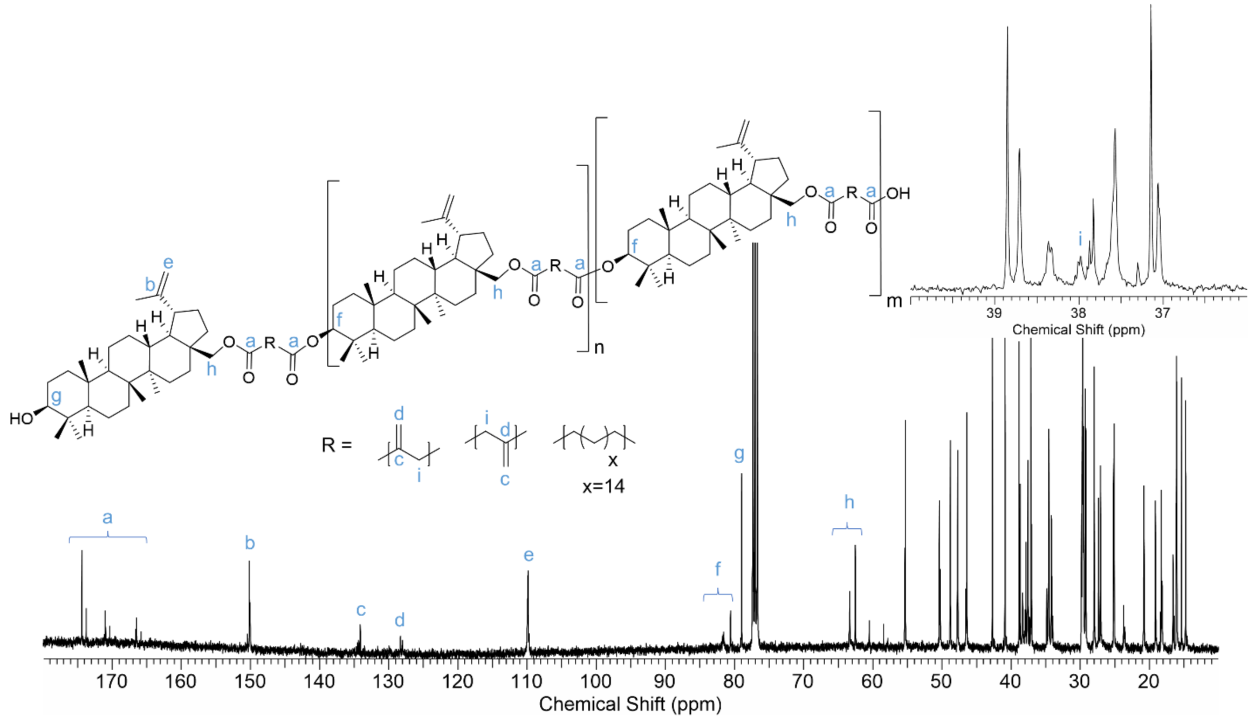

Figure S18. Representative ^{13}C-NMR spectrum for C18/IA75-TP

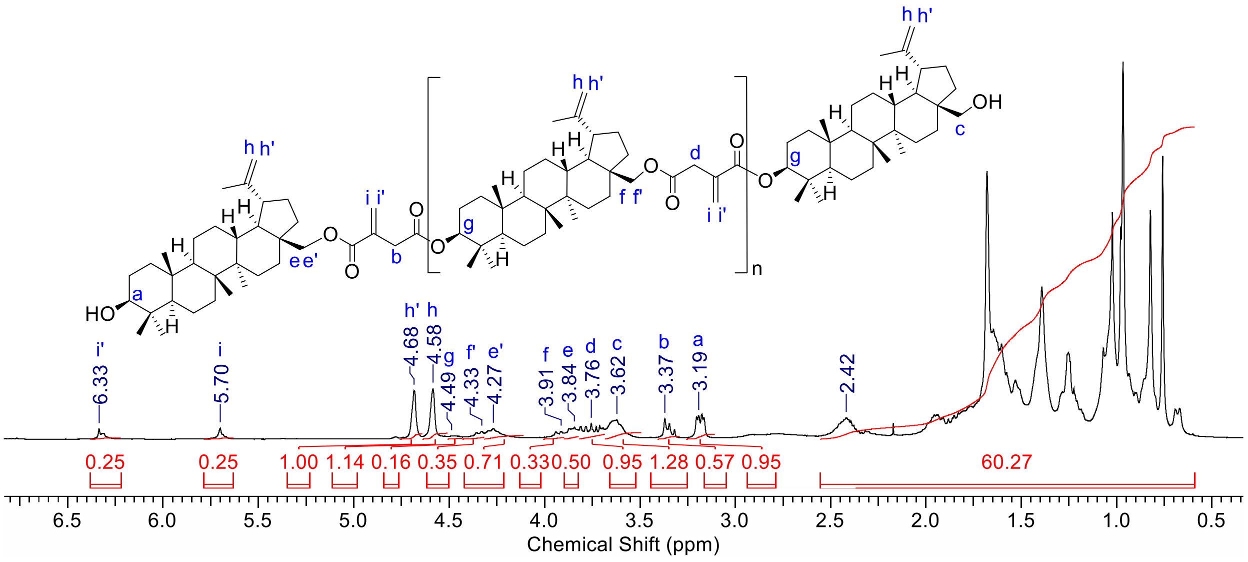

Figure S19. Representative ^{1}H-NMR spectrum for BDMI

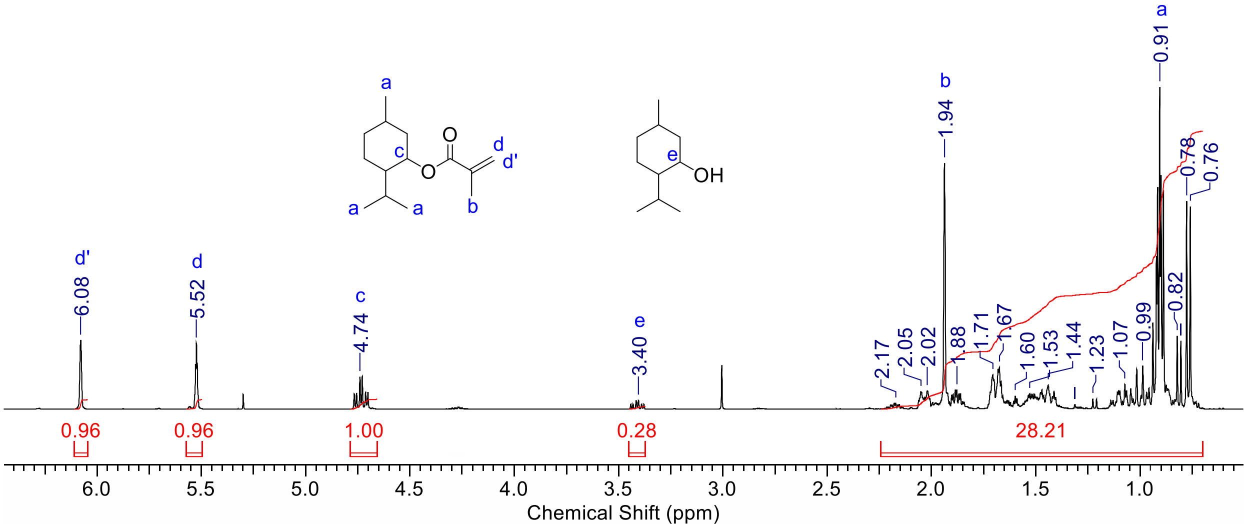

Figure S20. Representative ^{1}H-NMR spectrum for MenMA

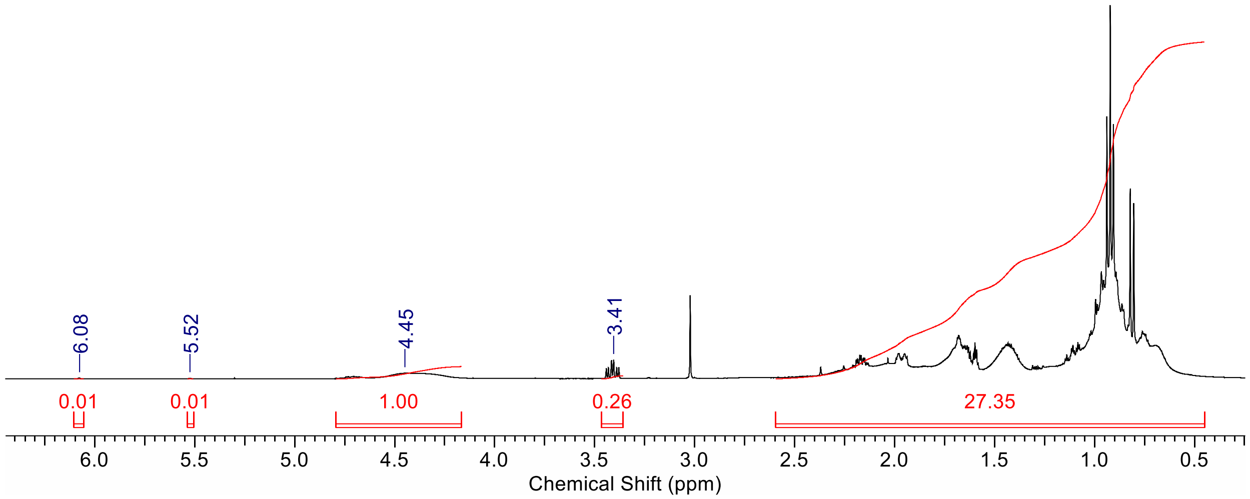

Figure S21. Representative ^{1}H-NMR spectrum for PMenMA

Table S2. Ratios of diacid to itaconic acid incorporated into polyesters according to ^{1}H-NMR peak assignments and product yields

Thermoplastic Polyester	Theoretical Diacid (mol)	Theoretical Itaconic Acid (mol)	Theoretical Diacid/Itaconic Acid Molar Ratio	Diacid/Itaconic Acid Molar Ratio from ^{1}H-NMR
C12/IA25-TP	75	25	3.0	8.5
C12/IA-TP	50	50	1.0	2.1
C12/IA75-TP	25	75	0.3	1.0
C18/IA25-TP	75	25	3.0	13.8
C18/IA-TP	50	50	1.0	2.5
C18/IA75-TP	25	75	0.3	1.1

6. BDMI Characterizations

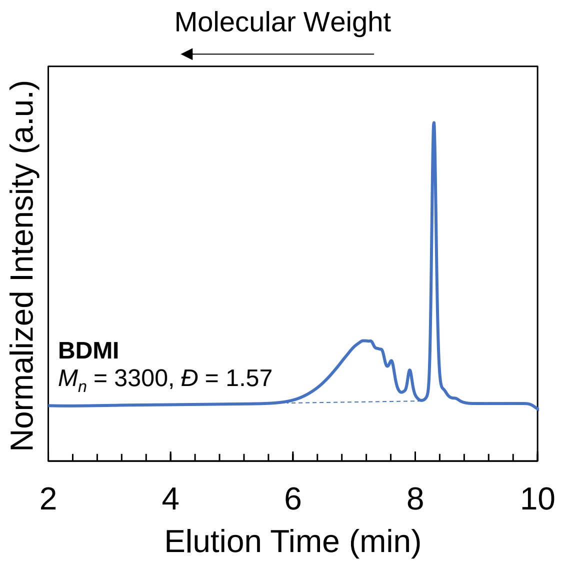

Figure S22. Representative APC chromatogram of BDMI

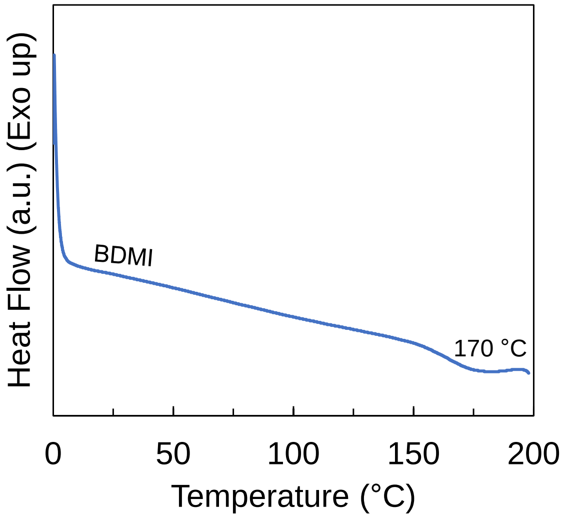

Figure S23. Representative DSC thermogram for BDMI

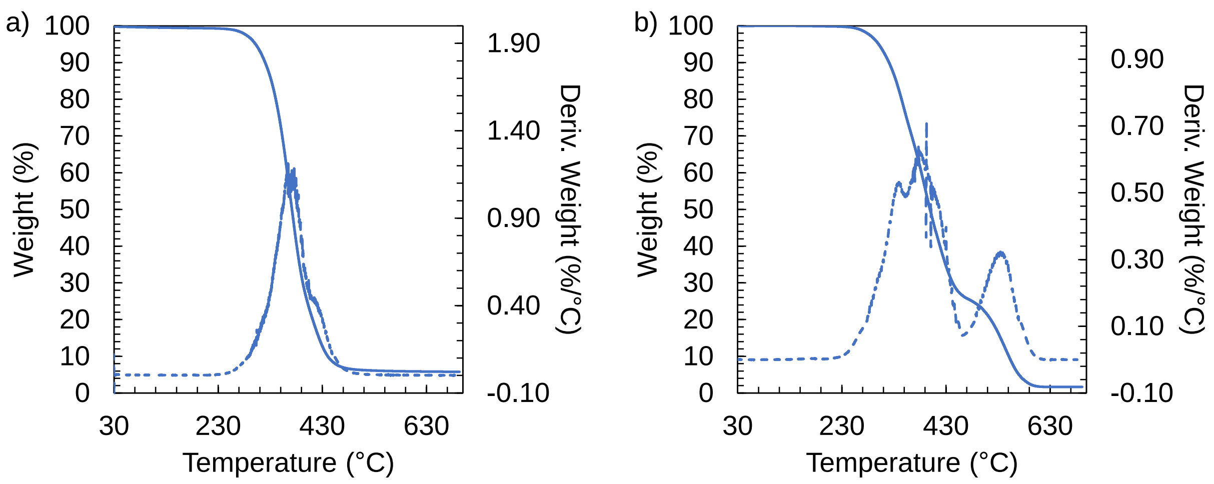

Figure S24. Representative TGA thermograms of BDMI and their first derivatives in a) N_2 and b) air environments

7. Additional APC Traces for Thermoplastic Precursors

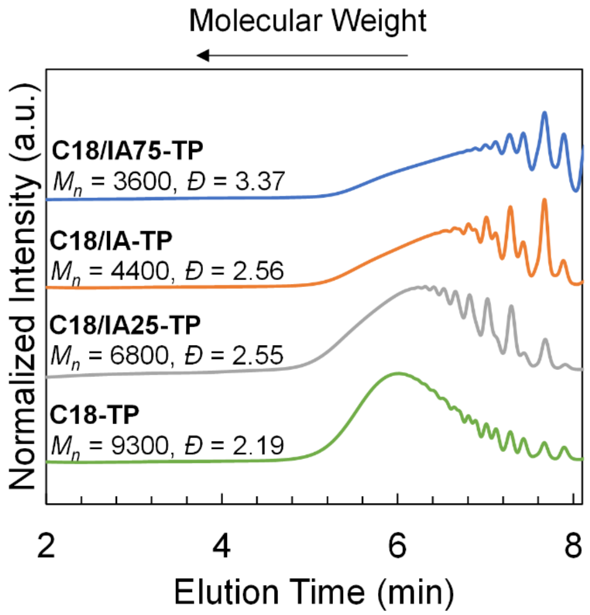

Figure S25. Representative APC traces of C18-based thermoplastic polyesters. Curves are normalized and offset for clarity.

8. Additional DSC Traces for Thermoplastic Precursors

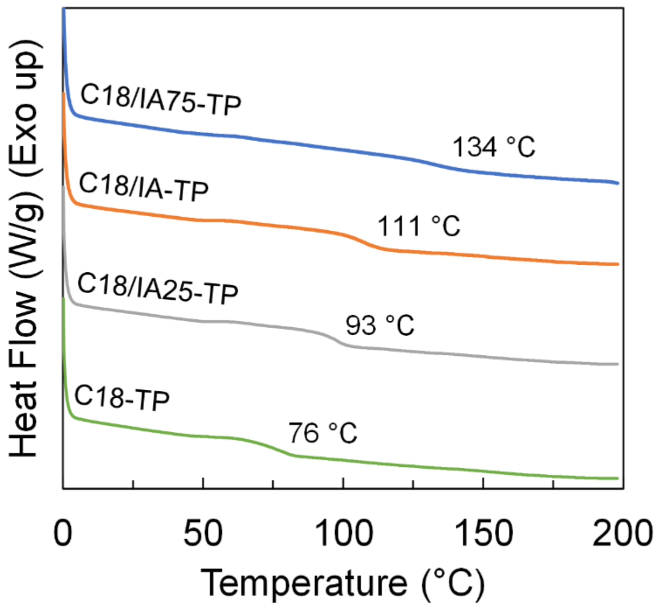

Figure S26. Representative DSC traces of C18-based thermoplastic polyesters (second heating ramp). Curves are normalized and offset for clarity.

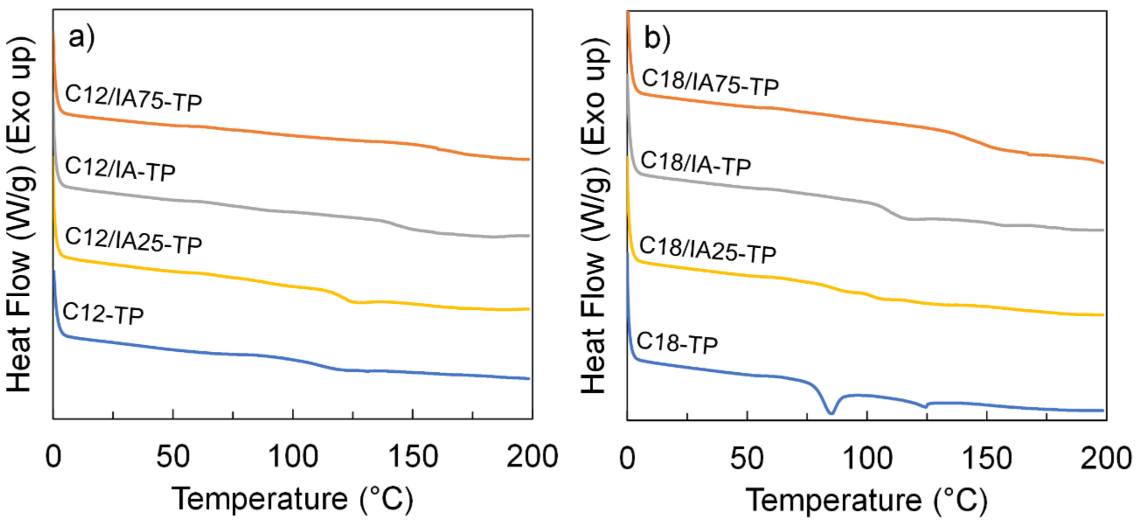

Figure S27. Representative DSC traces from the first heating ramp for a) C12-based and b) C18-based thermoplastic polyesters. Curves are normalized and offset for clarity.

9. TGA Results for Thermoplastic Precursors

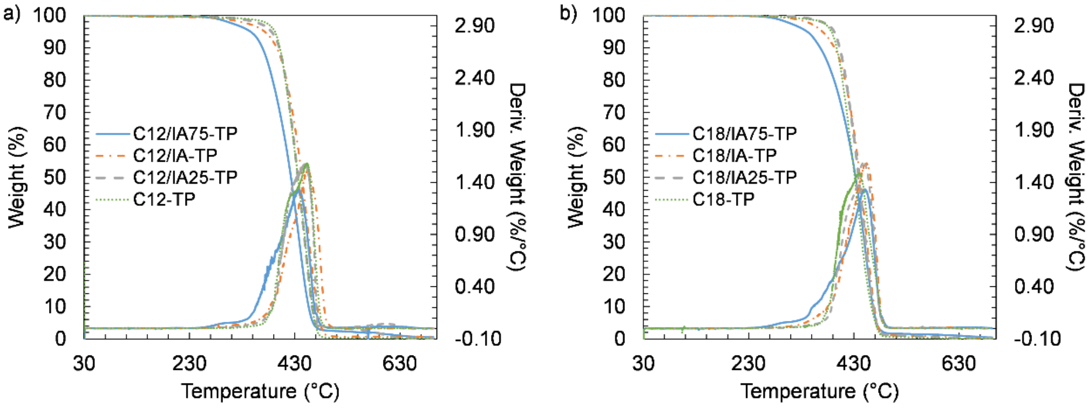

Figure S28. Representative TGA thermograms and their first derivatives in N_2 of a) C12-based and b) C18-based thermoplastic polyesters

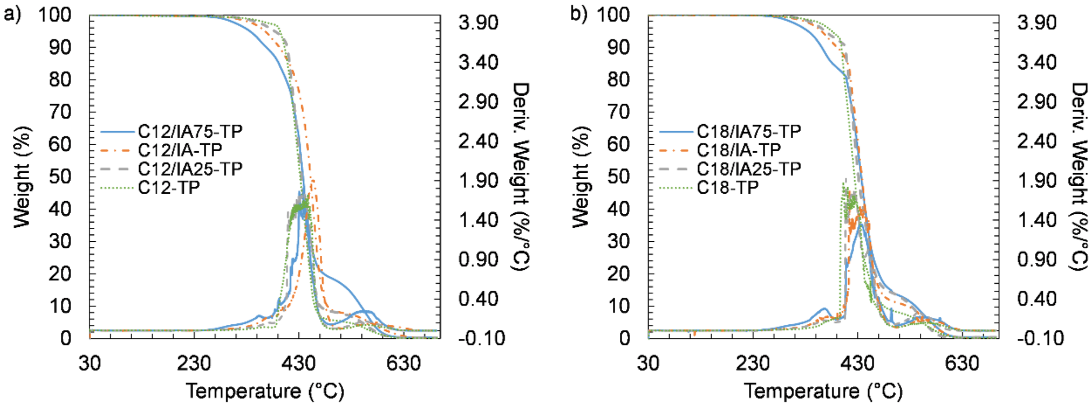

Figure S29. Representative TGA thermograms and their first derivatives in air of a) C12-based and b) C18-based thermoplastic polyesters

10. Rheology Results for PM Resins

Most resins appeared to consistently exhibit Newtonian behavior. A decrease in viscosity with increasing shear rate was observed for certain replicates of the 10 wt% C18/IA-meth and 25 wt% C18/IA-meth formulations, which is indicative of resin that either exhibits shear thinning behavior or contains a distribution of particles.[2] Qualitative observation of these resins confirmed that C18/IA-TP was not as well incorporated into the MenMA at the tested mixing conditions. The resin viscosities appeared to stabilize at 100 s^{-1} for all formulations. Therefore, Table S3 shows the viscosities of each resin at a shear rate of 100 s^{-1} for comparison.

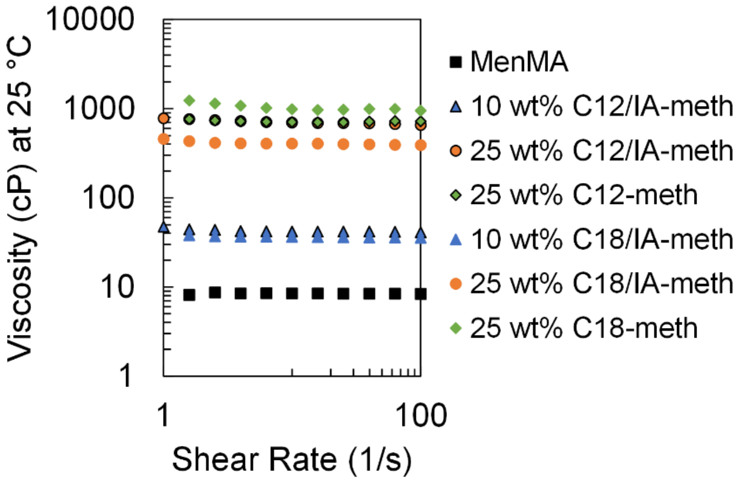

Figure S30. Viscosity overlay of representative results for liquid PM resins before UV curing

Table S3. Viscosities of liquid PM resins at 25 °C and a shear rate of 100 s^{-1}

Thermoplastic Polyester	Thermoplastic Polyester (wt%)	MenMA (wt%)	PM Resin	Viscosity at 25 °C (cP)
-	0	100	MenMA	8 ± < 1
C12/IA-TP	10	90	10 wt% C12/IA-meth	35 ± 6
C12/IA-TP	25	75	25 wt% C12/IA-meth	634 ± 36
C18/IA-TP	10	90	10 wt% C18/IA-meth	35 ± 5
C18/IA-TP	25	75	25 wt% C18/IA-meth	424 ± 46
C12-TP	25	75	25 wt% C12-meth	641 ± 72
C18-TP	25	75	25 wt% C18-meth	924 ± 68

11. Appearance of Cured PM Resins

The cured PMenMA homopolymer was transparent and colorless in appearance. The cured C12/IA-meth and C18/IA-meth resins appeared to increase in yellowing and opacity with increasing precursor content. This increase in opacity can be attributed to phase separation behavior during curing. The cured C12-meth and C18-meth resins appeared less glassy and exhibited a more cloudy appearance.

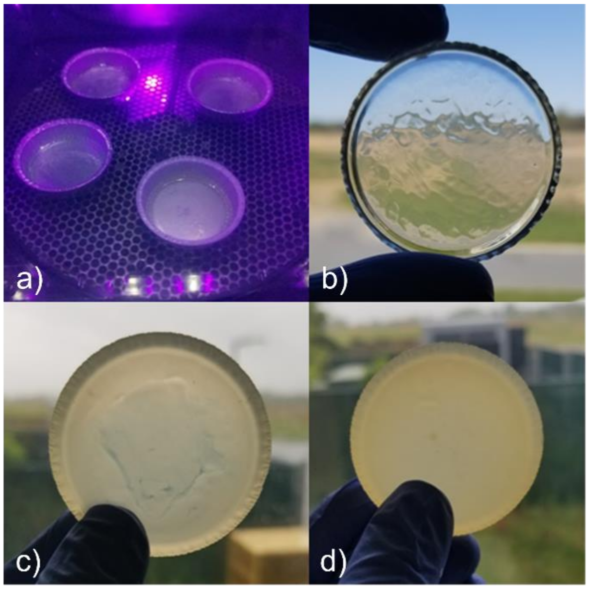

Figure S31. a) Samples in Formcure and cured b) PMenMA, c) 10 wt% C12/IA-meth, and d) 25 wt% C12/IA-meth cured resins

12. Additional FTIR Spectra for Cured and Uncured PM Resins and Thermoplastic Precursors

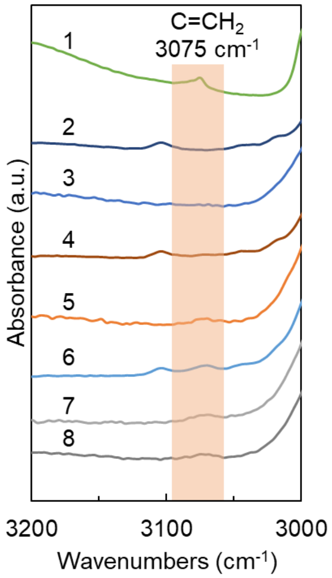

Figure S32. Representative FTIR spectra from 3000 cm^{-1} to 3200 cm^{-1} of 1) Betulin, 2) MenMA resin, 3) PMenMA, 4) 10 wt% C12/IA-meth uncured, 5) 10 wt% C12/IA-meth cured, 6) 25 wt% C12/IA-meth uncured, 7) 25 wt% C12/IA-meth cured and 8) 25 wt% C12-meth cured. Curves are normalized and offset for clarity.

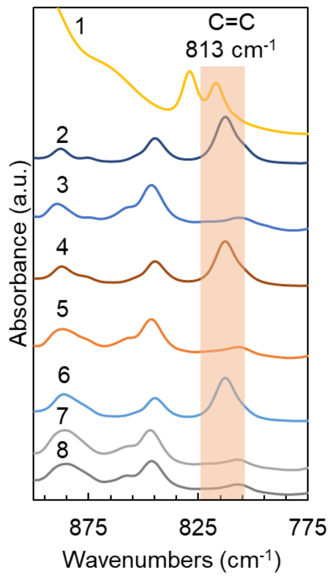

Figure S33. Representative FTIR spectra from 775 cm^{-1} to 900 cm^{-1} of 1) IA, 2) MenMA resin, 3) PMenMA, 4) 10 wt% C12/IA-meth uncured, 5) 10 wt% C12/IA-meth cured, 6) 25 wt% C12/IA-meth uncured, 7) 25 wt% C12/IA-meth cured and 8) 25 wt% C12-meth cured. Curves are normalized and offset for clarity.

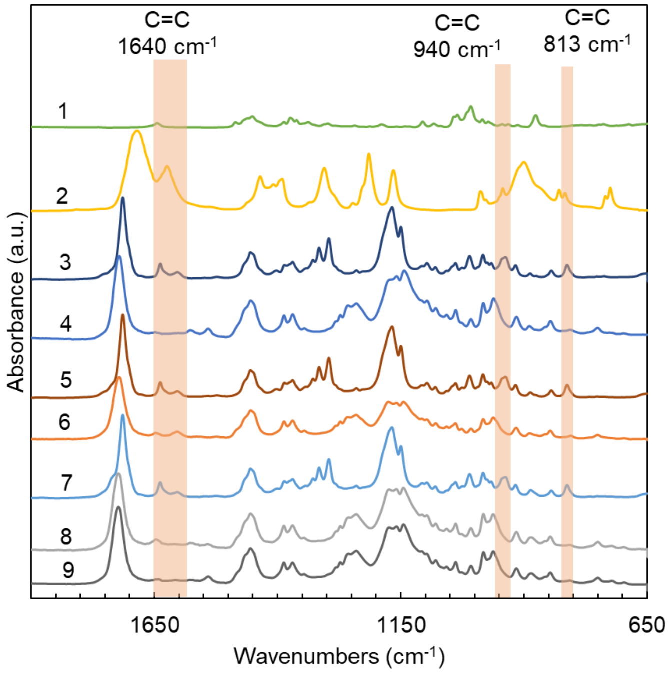

Figure S34. Representative FTIR spectra of 1) Betulin, 2) IA, 3) MenMA resin, 4) PMenMA, 5) 10 wt% C18/IA-meth uncured, 6) 10 wt% C18/IA-meth cured, 7) 25 wt% C18/IA-meth uncured, 8) 25 wt% C18/IA-meth cured and 9) 25 wt% C18-meth cured. Curves are normalized and offset for clarity.

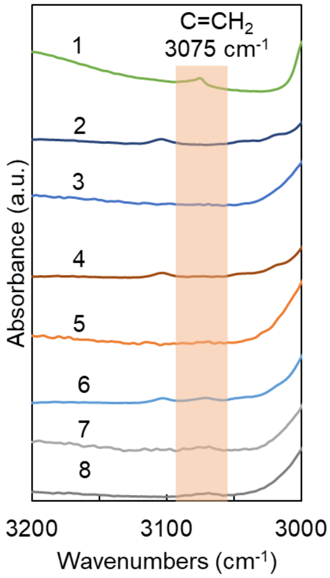

Figure S35. Representative FTIR spectra from 3000 to 3200 cm^{-1} of 1) Betulin, 2) MenMA resin, 3) PMenMA, 4) 10 wt% C12/IA-meth uncured, 5) 10 wt% C12/IA-meth cured, 6) 25 wt% C12/IA-meth uncured, 7) 25 wt% C12/IA-meth cured and 8) 25 wt% C12-meth cured. Curves are normalized and offset for clarity.

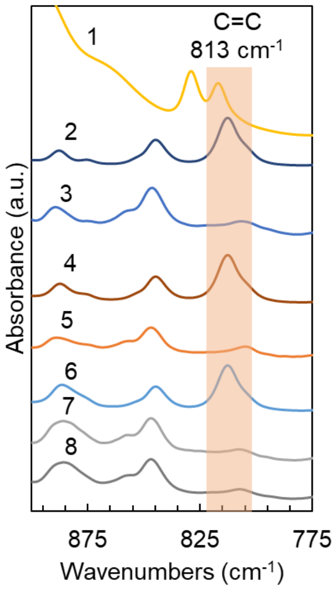

Figure S36. Representative FTIR spectra from 775 cm^{-1} to 900 cm^{-1} of 1) IA, 2) MenMA resin, 3) PMenMA, 4) 10 wt% C12/IA-meth uncured, 5) 10 wt% C12/IA-meth cured, 6) 25 wt% C12/IA-meth uncured, 7) 25 wt% C12/IA-meth cured and 8) 25 wt% C12-meth cured. Curves are normalized and offset for clarity.

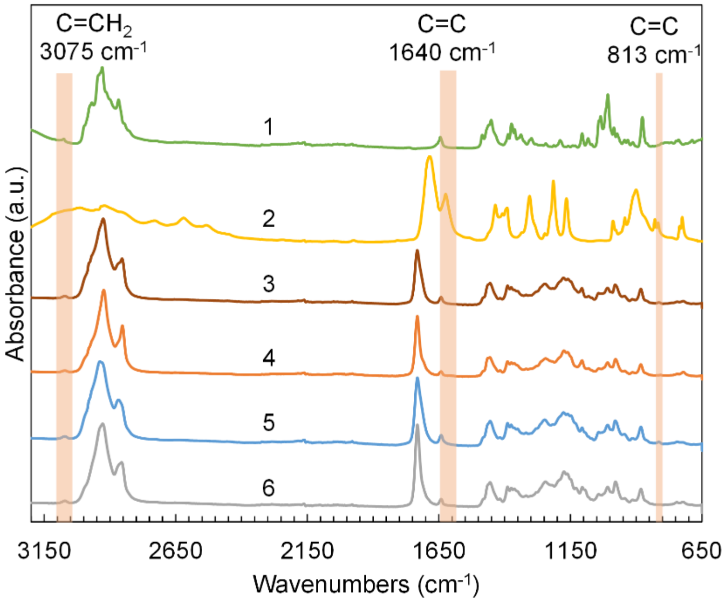

Figure S37. Representative FTIR spectra of 1) Betulin, 2) IA, 3) C18/IA-TP, 4) C18-TP, 5) C12/IA-TP, 6) C12-TP. Curves are normalized and offset for clarity.

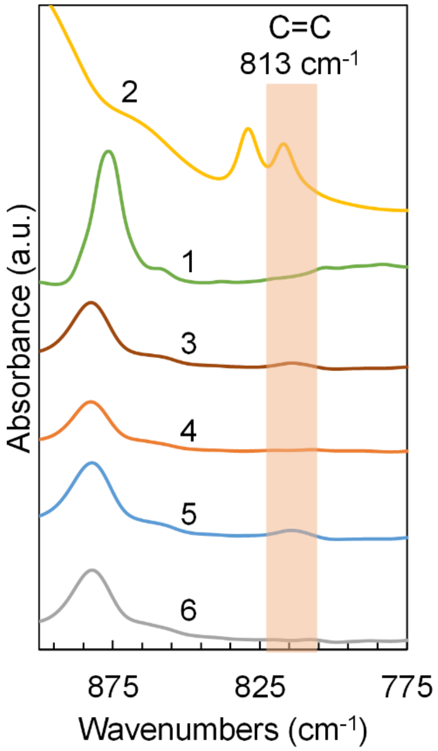

Figure S38. Representative FTIR spectra from 775 cm^{-1} to 900 cm^{-1} of 1) Betulin, 2) IA, 3) C18/IA-TP, 4) C18-TP, 5) C12/IA-TP, 6) C12-TP. Curves are normalized and offset for clarity.

13. DSC and Gel Content Results for PM Cured Resins

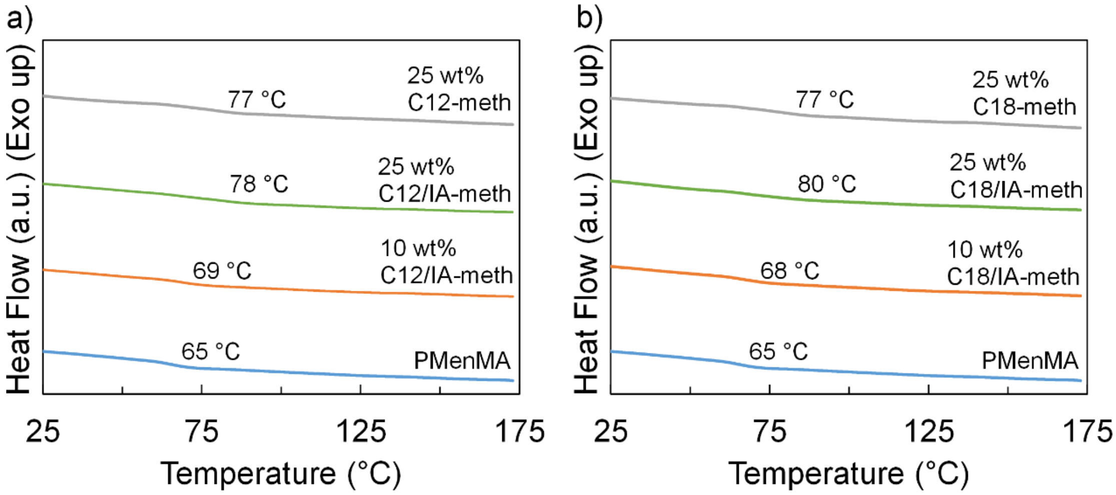

Figure S39. Representative DSC thermograms of a) C12-based and b) C18-based sequentially cured PM resins, shifted vertically for clarity. Second heating ramps are shown.

Table S4. DSC and gel content results for PM cured resins

Resin	T_g, DSC (°C)	Gel Content (%)
PMenMA	65 ± 1	0
10 wt% C12/IA-meth	69 ± 1	84 ± 16
25 wt% C12/IA-meth	78 ± 1	93 ± 6
25 wt% C12-meth	77 ± 2	-
10 wt% C18/IA-meth	68 ± 1	89 ± 7
25 wt% C12/IA-meth	80 ± 8	89 ± 5
25 wt% C18-meth	77 ± 1	-

14. TGA Results for PM Cured Resins and Cured PMenMA

Table S5. TGA results for cured PM resins and PMenMA

Resin	N_2				Air			
	**IDT (°C)**	T_{50} **(°C)**	T_{max} **(°C)**	**Char Yield* (%)**	**IDT (°C)**	T_{50} **(°C)**	T_{max} **(°C)**	**Char Yield* (%)**
PMenMA	195 ± 8	299 ± 9	302 ± 25	0.64 ± 0.37	197 ± 6	259 ± 8	259 ± 6	0.26 ± 0.28
10 wt% C12/IA-meth	195 ± 2	339 ± 2	345 ± 2	0.90 ± 0.70	194 ± 2	318 ± 11	343 ± 2	0.23 ± 0.07
25 wt% C12/IA-meth	208 ± 3	350 ± 1	345 ± 2	1.30 ± 0.33	203 ± 10	346 ± 3	346 ± 1	0.41 ± 0.22
25 wt% C12-meth	218 ± 5	345 ± 4	344 ± 2	0.54 ± 0.36	222 ± 5	337 ± 4	344 ± 1	0.31 ± 0.35
10 wt% C18/IA-meth	195 ± 3	335 ± 11	344 ± 2	0.89 ± 0.61	197 ± 3	322 ± 7	344 ± 4	0.11 ± 0.06
25 wt% C18/IA-meth	203 ± 4	349 ± 2	347 ± 2	1.16 ± 0.08	204 ± 8	345 ± 2	346 ± 1	0.28 ± 0.10
25 wt% C18-meth	212 ± 7	348 ± 2	347 ± 1	0.96 ± 0.17	211 ± 2	337 ± 2	345 ± 2	0.27 ± 0.14

*Char yield values determined at 700 °C

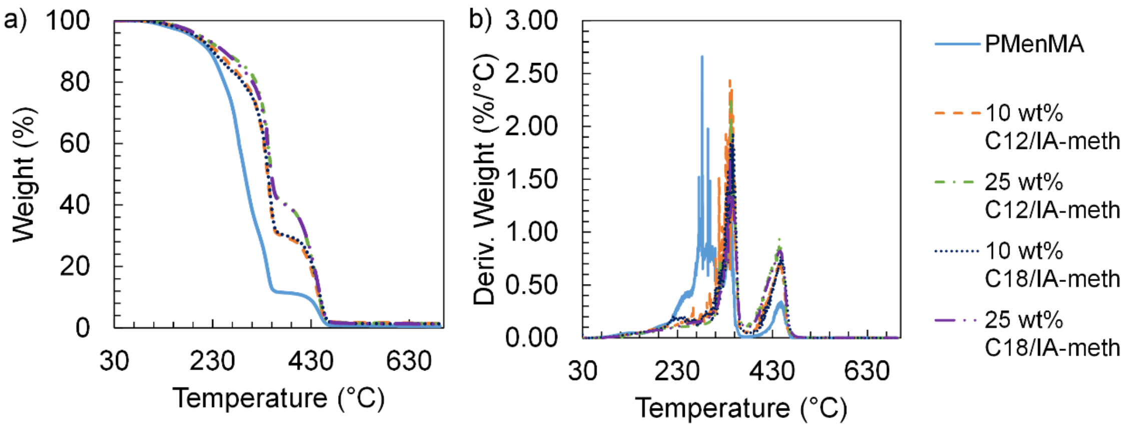

Figure S40. a) Representative TGA thermograms of cured PM resins and PMenMA in N_2 and b) the respective first derivative curves

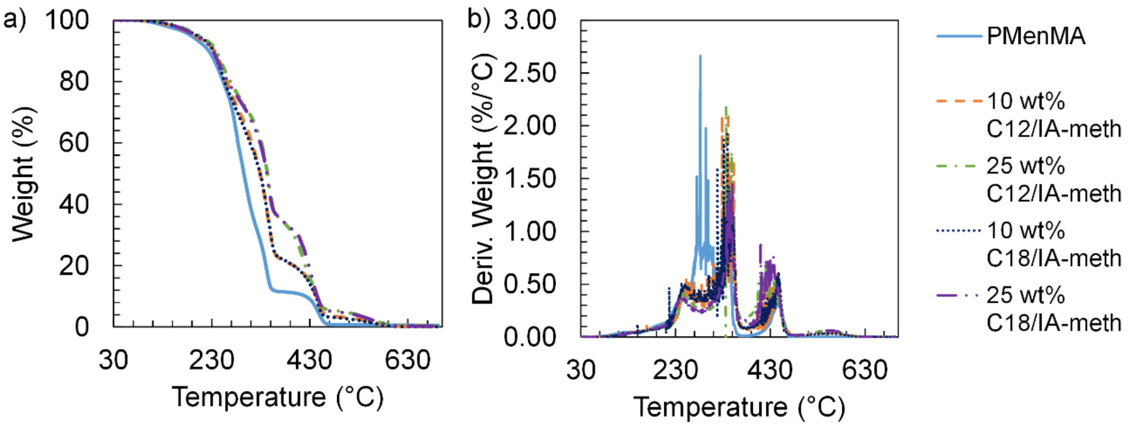

Figure S41. a) Representative TGA thermograms of cured PM resins and PMenMA in air and b) the respective first derivative curves

15. DSC Results for Bulk-cured Polyester Thermosets

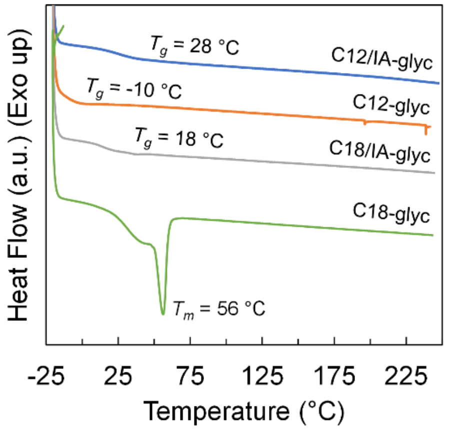

Figure S42. Representative DSC thermograms for the bulk-cured polyester thermosets. All curves are offset vertically for clarity. Curves show the second heating ramp for all samples.

16. Additional TGA Results for Bulk-cured Polyester Thermosets

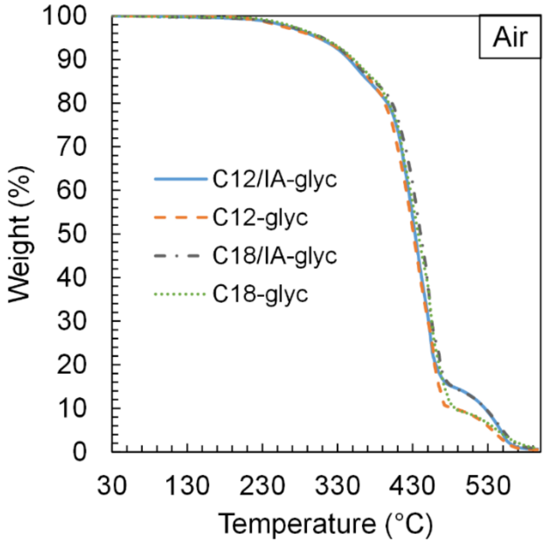

Figure S43. Representative TGA thermograms of the bulk-cured polyester thermosets in air

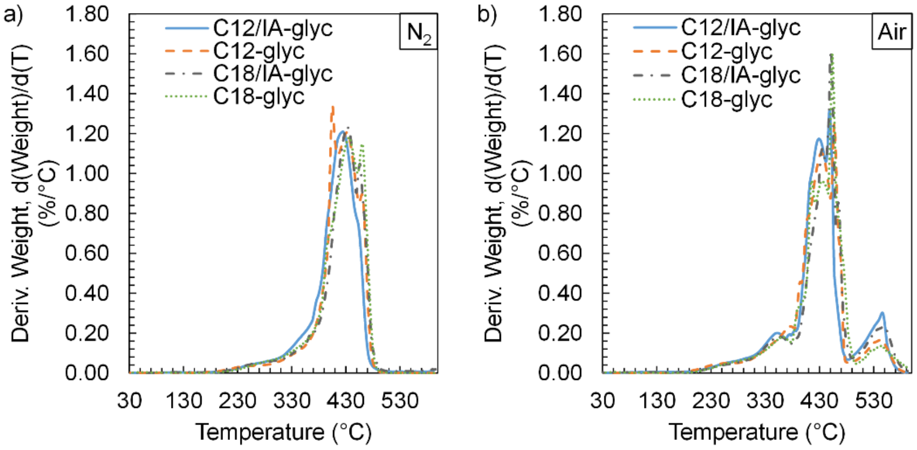

Figure S44. Representative TGA thermogram first derivatives of the bulk-cured polyester thermosets in a) N_2 and b) air environments

References

1. Achmad, F.; Yamane, K.; Quan, S.; Kokugan, T., Synthesis of polylactic acid by direct polycondensation under vacuum without catalysts, solvents and initiators. *Chemical Engineering Journal* **2009,** *151* (1-3), 342-350. DOI: 10.1016/j.cej.2009.04.014.

2. The influence of particles on suspension rheology. (accessed 01/09/2023).
